# Comparative effectiveness of pharmacological treatments for fetal growth restriction: a network meta-analysis

**DOI:** 10.3389/fphar.2026.1792975

**Published:** 2026-04-15

**Authors:** Yanting Wei, Ying Zhou, Xin Yu, Leilei Gong, Meng Wang, Xin Feng

**Affiliations:** 1 Pharmacy Department, Shijiazhuang Fourth Hospital, Shijiazhuang, China; 2 Department of Pharmacy, Peking University First Hospital, Beijing, China; 3 Beijing Obstetrics and Gynecology Hospital, Capital Medical University, Beijing Maternal and Child Healthcare Hospital, Beijing, China

**Keywords:** fetal growth restriction, intrauterine growth restriction, low molecular weight heparin, low-dose aspirin, network meta-analysis, pregnancy complications

## Abstract

**Background:**

Fetal growth restriction (FGR) is a common pregnancy complication associated with adverse maternal and fetal outcomes. Effective pharmacological interventions are essential for enhancing fetal growth and mitigating related complications. This study aimed to evaluate and compare the efficacy of various pharmacological treatments for FGR using a network meta-analysis (NMA).

**Objective:**

To systematically evaluate and compare the effectiveness of different pharmacological interventions for FGR via a network meta-analysis (NMA).

**Strategy:**

A comprehensive literature search was performed in PubMed, Medline, Embase, PsycINFO, the Cochrane Central Register of Controlled Trials, and Web of Science. The search was updated through 31 January 2026, to ensure the inclusion of the most recent evidence.

**Selection Criteria:**

Eligible studies included singleton pregnancies at high risk of FGR. Studies were excluded if they involved multiple pregnancies, fetal genetic abnormalities, or maternal comorbidities such as drug or alcohol abuse.

**Data Collection and Analysis:**

A systematic review and network meta-analysis were conducted in strict accordance with the PRISMA (Preferred Reporting Items for Systematic Reviews and Meta-Analyses) guidelines to ensure methodological rigor.

**Main Results:**

Compared with the control group and low-dose aspirin (LDA) alone, low-molecular-weight heparin (LMWH) and the combination of LMWH + LDA significantly reduced the incidence of intrauterine growth restriction (IUGR) (odds ratio [OR] = 0.40, 95% confidence interval [CI] = 0.26–0.62; OR = 0.37, 95% CI = 0.15–0.93, respectively). Additionally, LMWH monotherapy significantly decreased the risk of several pregnancy complications, including preeclampsia (OR = 0.21, 95% CI = 0.05–0.93), preterm birth (OR = 0.61, 95% CI = 0.45–0.81), miscarriage (OR = 0.42, 95% CI = 0.19–0.91), and cesarean section (OR = 0.34, 95% CI = 0.18–0.67). The combination of LMWH + LDA significantly improved the live birth rate (OR = 7.08, 95% CI = 2.16–23.22) and reduced the incidence of preeclampsia (OR = 0.22, 95% CI = 0.08–0.59).

**Conclusion:**

LMWH and LMWH combined with LDA are effective in reducing IUGR, preventing preeclampsia, and improving live birth rates in high-risk pregnancies complicated by FGR. These findings provide robust evidence supporting the use of LMWH and LMWH + LDA as promising therapeutic options for the management of FGR.

**Clinical Trial Registration:**

PROSPERO registration: CRD420251142968.

## 1 Introduction

Fetal growth restriction (FGR) refers to a condition marked by insufficient fetal growth during pregnancy, commonly defined as an estimated fetal weight below the 10th percentile for gestational age (Melamed et al., 2021). FGR is a major and prevalent pregnancy complication that affects approximately 3%–10% of pregnancies globally, with variations attributed to study populations and diagnostic criteria (Lees et al., 2022). The etiology of FGR is multifactorial, encompassing placental insufficiency, maternal disorders, IUIs, and genetic factors. It has several adverse outcomes for mothers and fetuses. There is an increased risk of stillbirth, preterm birth, neonatal mortality, and long-term developmental complications for the child and neurodevelopmental outcomes, including cognitive and motor delays, in affected pregnancies. The likelihood of complications, such as preeclampsia, cesarean delivery, and postpartum hemorrhage, is high for mothers (Figueras and Gratacós, 2014; Malacova et al., 2018). Beyond its medical implications, is a substantial economic burden on healthcare systems due to the need for intensive monitoring, preterm delivery, and long-term care for affected infants (Rakhanova et al., 2023). Effective FGR management is crucial because it can significantly reduce the risk of adverse outcomes, improve maternal and neonatal health, and potentially reduce healthcare costs associated with these pregnancies.

Currently, several pharmacological treatments, including anticoagulants, antiplatelet agents, and other drugs aimed at improving placental function, are used to manage FGR. Among these, low-dose aspirin (LDA), low molecular weight heparin (LMWH), and unfractionated heparin (UFH) are commonly employed (Melamed et al., 2021; Lees et al., 2022). These treatments seek to improve uteroplacental blood flow and reduce thrombotic complications, potentially improving fetal growth. However, while these treatments show some promise, their effectiveness remains inconclusive, as results from individual studies are often conflicting. Additionally, the variety of treatment approaches complicates decision-making, as no single drug has been universally accepted as the most effective for treating FGR (Figueras and Gratacós, 2014). Although some studies suggest these pharmacological interventions may improve fetal growth, systematic reviews and meta-analyses have demonstrated inconsistent findings across various settings (Malacova et al., 2018). Despite these promising avenues, high-quality evidence is insufficient to definitively recommend any specific therapeutic approach. A recent meta-analysis highlighted the efficacy of certain treatments but did not address the comparative effectiveness of different pharmacological strategies (Rakhanova et al., 2023). The lack of robust, comparative evidence remains a significant gap in the literature, particularly concerning the effectiveness of different drug regimens in improving FGR outcomes.

This study aims to address this gap by performing a network meta-analysis (NMA) to evaluate and compare the effectiveness of various pharmacological treatments for FGR. Unlike traditional pairwise meta-analyses, NMA allows for the simultaneous comparison of multiple treatments, even if some treatments have not been directly compared in individual studies (Leucht et al., 2016). The primary objective of this research is to provide a comprehensive analysis of available treatment options, helping to identify the most effective pharmacological strategies for managing FGR. This will not only inform clinical decision-making but also contribute to the optimization of treatment protocols, ultimately improving maternal and fetal health outcomes in pregnancies complicated by FGR. By synthesizing evidence from a diverse range of studies, this analysis will offer valuable insights into the comparative effectiveness of different pharmacological interventions, addressing current shortcomings in the literature and providing a more nuanced understanding of their potential benefits.

## 2 Methods and analysis

**FIGURE 1:**
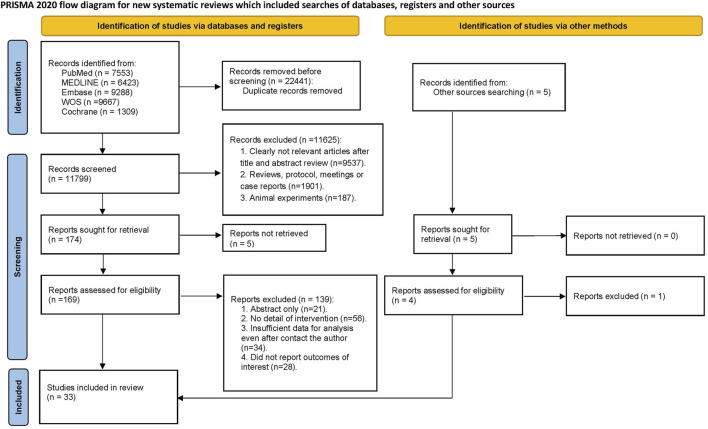
PRISMA Flow diagram of the search process for studies.

This systematic review and network meta-analysis was conducted following the guidelines outlined in the Preferred Reporting Items for Systematic Reviews and Meta-Analyses (PRISMA) 2020 statement and its extension for network meta-analysis in healthcare interventions (PRISMA-NMA)​ approval or consent for this meta-analysis was not required (Hutton et al., 2015; Page et al., 2021), as this study was based on publicly available data from previously published studies (Figure 1).

### 2.1 Type of patients

The study population consisted of singleton pregnancies at high risk of fetal growth restriction (FGR), defined as women meeting at least one of the following criteria: a history of FGR in previous pregnancies, history of late pregnancy loss or recurrent early pregnancy loss, hypertensive disorders during pregnancy, or thrombophilic conditions (including both inherited and acquired thrombophilia).

### 2.2 Type of studies

Studies eligible for inclusion were randomized controlled trials (RCTs) and observational cohort studies (both prospective and retrospective) published in English.

A mixed-design network meta-analysis (NMA) was pre-specified for this study to synthesize evidence from both RCTs and observational cohort studies. Studies involving multiple pregnancies, fetal genetic abnormalities, or maternal conditions such as drug or alcohol abuse were excluded (due to fundamentally different pathophysiology, risk profiles, and management compared to singleton pregnancies, which could introduce substantial heterogeneity).

RCTs are the gold standard for evaluating causal treatment effects, providing the highest level of evidence for pharmacological intervention efficacy. Observational cohort studies complement RCTs by reflecting real-world clinical practice. The integration of both study designs in mixed-design NMA was deemed necessary to maximize the available evidence base, enhance the generalizability of findings.

### 2.3 Types of interventions

The interventions evaluated included unfractionated heparin (UFH), low molecular weight heparin (LMWH), low-dose aspirin (LDA), other antiplatelet agents, and pharmacological treatments such as phosphodiesterase inhibitors, nitric oxide donors, and statins, either alone or in combination. Studies comparing these interventions to placebo, no treatment, or standard care without the investigated pharmacological interventions (control groups) were included.

### 2.4 Types of outcome measures

The primary outcome was FGR, as defined by the authors of each study. Secondary outcomes included preterm birth, placental abruption, fetal or neonatal death, live birth rates, and pregnancy complications (e.g., preeclampsia). Safety outcomes including bleeding complications, thrombocytopenia, and other adverse events were prespecified as observation indicators, while valid and comparable data for these safety outcomes were insufficient among included studies for further quantitative analysis.

### 2.5 Information source and search strategy

A comprehensive literature search was conducted in PubMed, Embase, Cochrane Central Register of Controlled Trials, and Web of Science. The search was updated as of 31 January 2026. Search terms included: “fetal growth restriction,” “intrauterine growth restriction,” “pharmacological interventions,” “heparin,” “aspirin,” and related keywords. The complete search strategy is detailed in Supplementary Material 1 and Figure 1.

### 2.6 Study selection

Two independent researchers screened titles, abstracts, and full-text articles against inclusion criteria. Discrepancies were resolved by discussion or adjudication by a third reviewer. Reference lists of relevant articles published in the last 5 years were also reviewed to ensure comprehensive inclusion.

### 2.7 Data Collection

Data on study characteristics (e.g., authors, publication year, patient demographics), intervention details, and outcomes were extracted independently by two reviewers using EndNote X9. Continuous variables were extracted as mean and standard deviation, and categorical variables as event counts and sample sizes. Missing data were requested from corresponding authors via email.

### 2.8 Risk of bias assessment

The Cochrane Risk of Bias Tool (RoB 2) was used for RCTs, assessing domains such as sequence generation and blinding. For non-randomized studies, the Newcastle-Ottawa Scale (NOS) was applied, with a score of ≥5 indicating high quality. Two reviewers performed assessments independently, with disagreements resolved by consensus.

### 2.9 Data analysis

This network meta-analysis was performed employing a frequentist statistical framework utilizing a random-effects model to accommodate heterogeneity both within and across studies. All analyses were conducted using Stata version 17.0 (StataCorp LLC, Texas, United States of America) with implementation via the specialized networkpackage. The analytical approach was predicated on the consistency assumption, presupposing agreement between direct and indirect evidence, with validation through the node-splitting methodology for assessing local inconsistency and design-by-treatment interaction models for evaluating global inconsistency. Between-study heterogeneity was quantified using the I^2^ statistic with conventional thresholds (25%, 50%, and 75% for low, moderate, and high heterogeneity, respectively), while between-study variance (τ^2^) was estimated via restricted maximum likelihood (REML). Trials with multiple arms were incorporated using a multivariate covariance structure to account for within-trial correlations.

Treatment effects were expressed as mean differences (MDs) with corresponding 95% confidence intervals (CIs) for continuous outcomes and odds ratios (ORs) for dichotomous outcomes. The relative ranking of interventions was determined using the surface under the cumulative ranking curve (SUCRA) metric, wherein higher values indicate superior performance. Potential publication bias was investigated through visual inspection of funnel plots and formal statistical testing using Egger’s regression test, with a significance threshold of p < 0.05 (Chaimani et al., 2013). The methodological reporting rigorously adheres to the PRISMA-NMA guidelines and aligns with contemporary methodological standards to ensure analytical transparency and reproducibility. Key software commands executed included network mapfor network geometry visualization, mvmetafor effect size estimation under the REML constraint, and network sidesplitfor inconsistency diagnostics. Given the mixed-design study integration and the exploratory nature of the comparative treatment analyses, a pre-specified GRADE-for-NMA certainty-of-evidence assessment was not conducted for the network estimates generated in this analysis.

## 3 Results

### 3.1 Characteristics of included studies

The initial electronic search identified 34,240 records. After removing 22,441 duplicate entries, 11,799 articles underwent title and abstract screening. A total of 11,625 studies were excluded based on title and abstract, and 169 articles were further assessed for full-text eligibility. Ultimately, 33 studies were included in this systematic review and network meta-analysis, comprising 5,858 singleton pregnancies at high risk for FGR (Abdi et al., 2020; Badawy et al., 2008; Beaufils et al., 1985; Chen et al., 2023; Cowchock and Reece, 1997; de Vries et al., 2012; Dugalic et al., 2019; Fouda et al., 2011; Furuhashi et al., 2021; Gris et al., 2011; Gris et al., 2004; Groom et al., 2017; Haddad et al., 2016; Huai et al., 2021; Kupferminc M. et al., 2011; Kupferminc M. J. et al., 2011; Kutteh, 1996; Landman et al., 2022; Martinelli et al., 2012; Mello et al., 2005; Mirzamoradi et al., 2023; Mohamed and Asjmefsj, 2014; Odibo et al., 2015; Pasquier et al., 2015; Rasmark Roepke et al., 2019; Schleussner et al., 2015; Sergio et al., 2006; Shaaban et al., 2017; Talari et al., 2014; Tulppala et al., 1997; Uzan et al., 1991; van Hoorn et al., 2016; Yuksel et al., 2014). The detailed characteristics of the included studies are provided in Supplementary Material 2. Among the 33 studies, 25 were RCTs, 2 were non-randomized trials, 2 were retrospective cohort studies, and 4 were prospective cohort studies. These studies were published between 1985 and 2023, with a median publication year of 2014.

The interventions evaluated included LDA (18 studies), LMWH (13 studies), LMWH + LDA (7 studies), dipyridamole + LDA (2 studies), UFH + LDA (2 studies), tadalafil (1 study), and controls (23 studies).

### 3.2 Network meta-analysis

Local and global inconsistency tests indicate that no significant inconsistency was found between direct and indirect evidence in the network, supporting the consistency of treatment comparisons.

#### 3.2.1 Fetal and neonatal outcomes

##### 3.2.1.1 Birth weight

**FIGURE 2:**
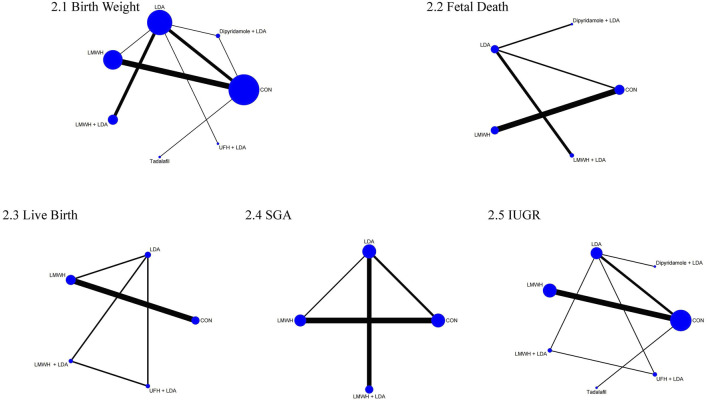
Network of treatment comparisons for fetal and neonatal outcomes. 2.1 birth weight, 2.2 fetal death, 2.3 live birth, 2.4 SGA, 2.5 IUGR.

**FIGURE 3:**
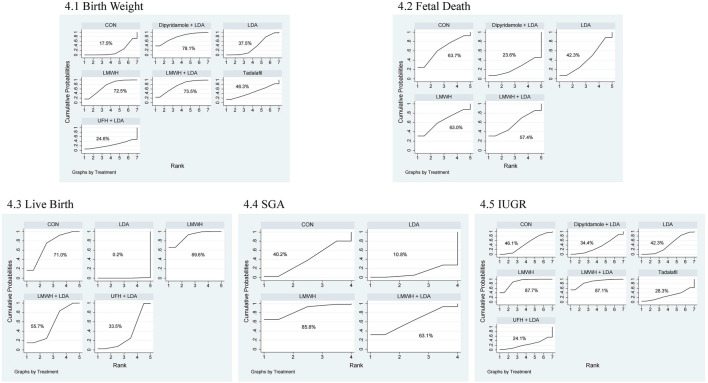
Ranking of treatment strategies based on probability of impact on fetal and neonatal outcomes. 4.1 birth weight, 4.2 fetal death, 4.3 live birth, 4.4 SGA, 4.5 IUGR.

The network meta-analysis for birth weight included 24 studies with 3,490 participants, comparing the effects of various pharmacological treatments. Figure 2 illustrates the direct comparisons and sample size distribution across different treatment groups. Based on the Surface Under the Cumulative Ranking (SUCRA) values (Figure 3), the top three treatments for increasing neonatal birth weight were dipyridamole + LDA (78.1%), LMWH + LDA (73.5%), and LMWH (72.5%). However, as shown in Table 1 no significant differences were found between the treatments.

**TABLE 1:** Birth weight: mean differences and 95% confidence intervals from pairwise comparisons.

Dipyridamole + LDA	​	​	​	​	​	​
43.05 (−350.43,436.53)	LMWH + LDA	​	​	​	​	​
50.82 (−299.34,400.98)	7.77 (−296.65,312.19)	LMWH	​	​	​	​
185.77 (−350.27,721.81)	142.72 (−367.41,652.85)	134.95 (−314.25,584.15)	Tadalafil	​	​	​
211.14 (−111.71,534.00)	168.09 (−56.62,392.80)	160.33 (−44.85,365.50)	25.37 (−432.64,483.39)	LDA	​	​
353.14 (−275.96,982.25)	310.09 (−274.74,894.93)	302.33 (−275.28,879.93)	167.37 (−540.66,875.41)	142.00 (−397.94,681.94)	UFH + LDA	​
306.77 (−18.67,632.22)	263.72 (−17.01,544.45)	255.95 (113.28,398.62)	121.00 (−304.94,546.94)	95.63 (−72.75,264.01)	−46.37 (−611.96,519.21)	CON

##### 3.2.1.2 Fetal death

The network meta-analysis for fetal death included 8 studies with 1,065 participants. Figure 2 shows the direct comparisons and sample size distribution. The SUCRA ranking (Figure 3) indicated that the top three treatments for reducing fetal death were controls (63.7%), LMWH (63.0%), and LMWH + LDA (57.4%). However, Table 2 reveals no significant differences between the treatments.

**TABLE 2:** Fetal death: mean differences and 95% confidence intervals from pairwise comparisons.

CON	​	​	​	​
1.01 (0.23,4.50)	LMWH	​	​	​
0.72 (0.01,45.98)	0.71 (0.01,58.79)	LMWH + LDA	​	​
0.41 (0.01,11.46)	0.41 (0.01,15.55)	0.57 (0.05,6.99)	LDA	​
0.19 (0.00,11.64)	0.19 (0.00,14.93)	0.27 (0.01,8.60)	0.46 (0.04,5.19)	Dipyridamole + LDA

##### 3.2.1.3 Live birth

The live birth network meta-analysis involved 8 studies with 1,228 participants. Figure 2 presents the direct comparisons and sample size distribution. SUCRA values (Figure 3) showed that the top three treatments for increasing live birth rates were LMWH (89.6%), controls (71.0%), and LMWH + LDA (55.7%). As shown in Table 3, LMWH (OR = 15.55, 95% CI = 5.49, 43.99), controls (OR = 12.63, 95% CI = 4.00, 39.87), LMWH + LDA (OR = 7.08, 95% CI = 2.16, 23.22), and unfractionated heparin + LDA (OR = 4.20, 95% CI = 1.31, 13.44) significantly increased live birth rates compared to LDA.

**TABLE 3:** Live birth: mean differences and 95% confidence intervals from pairwise comparisons.

LMWH	​	​	​	​
1.23 (0.75,2.01)	CON	​	​	​
2.20 (0.45,10.65)	1.78 (0.34,9.32)	LMWH + LDA	​	​
3.70 (0.78,17.59)	3.00 (0.59,15.41)	1.68 (0.54,5.21)	UFH + LDA	​
15.55 (5.49,43.99)	12.63 (4.00,39.87)	7.08 (2.16,23.22)	4.20 (1.31,13.44)	LDA

##### 3.2.1.4 Small for gestational Age (SGA)

The network meta-analysis for SGA included 12 studies with 2,115 participants. Figure 2 illustrates the direct comparisons and sample size distribution. According to the SUCRA ranking (Figure 3), the top three treatments for reducing SGA occurrence were LMWH (85.8%), LMWH + LDA (63.1%), and controls (40.2%). However, Table 4 shows no significant differences between the treatments.

**TABLE 4:** SGA: mean differences and 95% confidence intervals from pairwise comparisons.

LMWH	​	​	​
0.78 (0.26,2.35)	LMWH + LDA	​	​
0.64 (0.36,1.12)	0.82 (0.29,2.32)	CON	​
0.46 (0.20,1.09)	0.60 (0.30,1.20)	0.73 (0.34,1.55)	LDA

##### 3.2.1.5 Intrauterine growth restriction (IUGR)

The IUGR network meta-analysis included 15 studies with 1,056 participants. Figure 2 displays the direct comparisons and sample size distribution. Based on the SUCRA ranking (Figure 3), the top three treatments for reducing IUGR occurrence were LMWH (87.7%), LMWH + LDA (87.1%), and controls (46.1%). Table 5 shows that LMWH significantly reduced IUGR compared to controls (OR = 0.40, 95% CI = 0.26, 0.62) and LDA (OR = 0.37, 95% CI = 0.15, 0.93).

**TABLE 5:** IUGR: mean differences and 95% confidence intervals from pairwise comparisons.

LMWH	​	​	​	​	​	​
1.11 (0.22,5.53)	LMWH + LDA	​	​	​	​	​
0.40 (0.26,0.62)	0.36 (0.08,1.70)	CON	​	​	​	​
0.37 (0.15,0.93)	0.33 (0.09,1.24)	0.92 (0.41,2.08)	LDA	​	​	​
0.32 (0.10,1.04)	0.29 (0.06,1.29)	0.79 (0.26,2.38)	0.86 (0.42,1.80)	Dipyridamole + LDA	​	​
0.24 (0.04,1.40)	0.22 (0.02,2.17)	0.60 (0.11,3.30)	0.65 (0.10,4.33)	0.76 (0.10,5.74)	Tadalafil	​
0.21 (0.03,1.62)	0.19 (0.03,1.20)	0.53 (0.07,3.84)	0.58 (0.09,3.51)	0.67 (0.09,4.69)	0.88 (0.06,12.03)	UFH + LDA

#### 3.2.2 Pregnancy and delivery outcomes

##### 3.2.2.1 Placental abruption

**FIGURE 4:**
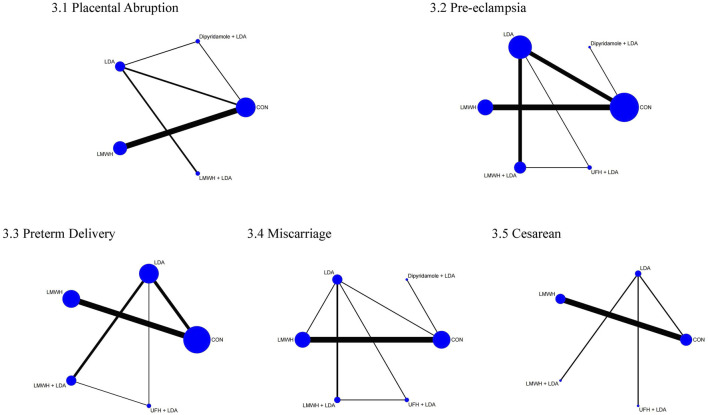
Network of treatment comparisons for pregnancy and delivery outcomes. 3.1 placental abruption, 3.2 pre-eclampsia, 3.3 preterm delivery, 3.4 miscarriage, 3.5 cesarean.

**FIGURE 5:**
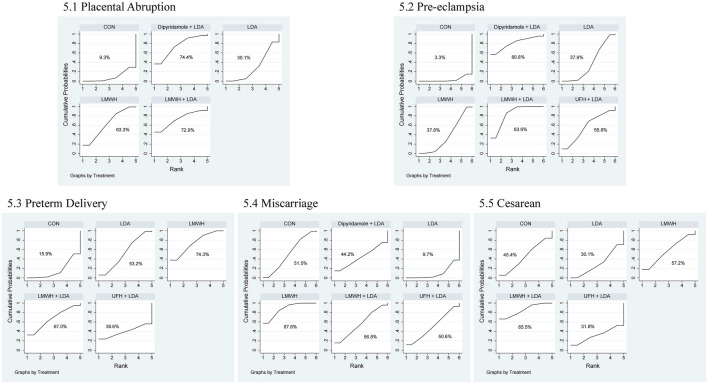
Ranking of treatment strategies based on probability of impact on pregnancy and delivery outcomes. 5.1 placental abruption, 5.2 pre-eclampsia, 5.3 preterm delivery, 5.4 miscarriage, 5.5 cesarean.

The network meta-analysis for placental abruption included 13 studies with 2,452 participants. Figure 4 displays the direct comparisons and sample size distribution. According to the SUCRA ranking (Figure 5), the top three treatments for reducing placental abruption were dipyridamole + LDA (74.4%), LMWH + LDA (72.9%), and LMWH (63.3%). Table 6 reveals that LMWH significantly reduced the incidence of placental abruption compared to controls (OR = 0.37, 95% CI = 0.14, 0.93).

**TABLE 6:** Placental abruption: odds ratios (OR) and 95% confidence intervals from pairwise comparisons.

Dipyridamole + LDA	​	​	​	​
1.10 (0.10,12.40)	LMWH + LDA	​	​	​
0.69 (0.11,4.23)	0.63 (0.06,6.72)	LMWH	​	​
0.38 (0.09,1.58)	0.34 (0.05,2.43)	0.54 (0.14,2.06)	LDA	​
0.25 (0.05,1.19)	0.23 (0.03,2.02)	0.37 (0.14,0.93)	0.68 (0.26,1.72)	CON

##### 3.2.2.2 Pre-eclampsia

The network meta-analysis for pre-eclampsia included 22 studies with 3,565 participants. Figure 4 shows the direct comparisons and sample size distribution. Based on the SUCRA ranking (Figure 5), the top three treatments for reducing pre-eclampsia were LMWH + LDA (83.6%), dipyridamole + LDA (80.8%), and unfractionated heparin + LDA (56.8%). Table 7 shows that LMWH + LDA significantly reduced pre-eclampsia compared to LDA (OR = 0.22, 95% CI = 0.08, 0.59), LMWH (OR = 0.21, 95% CI = 0.05, 0.93), and controls (OR = 0.10, 95% CI = 0.03, 0.35).

**TABLE 7:** Pre-eclampsia: odds ratios (OR) and 95% confidence intervals from pairwise comparisons.

LMWH + LDA	​	​	​	​	​
1.53 (0.05,47.21)	Dipyridamole + LDA	​	​	​	​
0.43 (0.06,3.25)	0.28 (0.01,13.26)	UFH + LDA	​	​	​
0.22 (0.08,0.59)	0.15 (0.01,3.86)	0.52 (0.07,3.96)	LDA	​	​
0.21 (0.05,0.93)	0.14 (0.01,3.63)	0.50 (0.05,5.00)	0.96 (0.33,2.84)	LMWH	​
0.10 (0.03,0.35)	0.06 (0.00,1.51)	0.22 (0.03,2.01)	0.43 (0.19,0.98)	0.45 (0.22,0.92)	CON

##### 3.2.2.3 Preterm delivery

The network meta-analysis for preterm delivery included 20 studies with 3,048 participants. Figure 4 presents the direct comparisons and sample size distribution. The SUCRA ranking (Figure 5) showed that the top three treatments for reducing preterm delivery were LMWH (74.3%), LMWH + LDA (67.0%), and LDA (53.2%). Table 8 shows that LMWH significantly reduced preterm delivery compared to controls (OR = 0.61, 95% CI = 0.45, 0.81).

**TABLE 8:** Preterm delivery: odds ratios (OR) and 95% confidence intervals from pairwise comparisons.

LMWH	​	​	​	​
0.96 (0.40,2.30)	LMWH + LDA	​	​	​
0.83 (0.51,1.35)	0.87 (0.42,1.79)	LDA	​	​
0.66 (0.13,3.30)	0.69 (0.15,3.08)	0.79 (0.17,3.68)	UFH + LDA	​
0.61 (0.45,0.81)	0.63 (0.28,1.45)	0.73 (0.50,1.08)	0.92 (0.19,4.51)	CON

##### 3.2.2.4 Miscarriage

The network meta-analysis for miscarriage included 14 studies with 2044 participants. Figure 4 displays the direct comparisons and sample size distribution. According to the SUCRA ranking (Figure 5), the top three treatments for reducing miscarriage rates were LMWH (87.6%), LMWH + LDA (56.8%), and controls (51.5%). Table 9 shows that LMWH significantly reduced miscarriage rates compared to controls (OR = 0.42, 95% CI = 0.19, 0.91) and LDA (OR = 0.13, 95% CI = 0.03, 0.53).

**TABLE 9:** Miscarriage: odds ratios (OR) and 95% confidence intervals from pairwise comparisons.

LMWH	​	​	​	​	​
0.45 (0.05,3.76)	LMWH + LDA	​	​	​	​
0.42 (0.19,0.91)	0.93 (0.11,7.72)	CON	​	​	​
0.38 (0.04,3.37)	0.85 (0.16,4.43)	0.91 (0.10,8.08)	UFH + LDA	​	​
0.32 (0.03,3.55)	0.71 (0.03,15.93)	0.77 (0.08,7.44)	0.84 (0.04,19.74)	Dipyridamole + LDA	​
0.13 (0.03,0.53)	0.29 (0.06,1.43)	0.31 (0.07,1.30)	0.34 (0.06,1.81)	0.41 (0.03,5.95)	LDA

##### 3.2.2.5 Cesarean section

The network meta-analysis for cesarean section included 8 studies with 1,305 participants. Figure 4 shows the direct comparisons and sample size distribution. According to the SUCRA ranking (Figure 5), the top three treatments for reducing cesarean section rates were LMWH + LDA (85.5%), LMWH (57.2%), and controls (45.4%). Table 10 shows that LMWH + LDA significantly reduced cesarean section rates compared to LDA (OR = 0.34, 95% CI = 0.18, 0.67).

**TABLE 10:** Cesarean section: odds ratios (OR) and 95% confidence intervals from pairwise comparisons.

LMWH + LDA	​	​	​	​
0.53 (0.09,3.23)	LMWH	​	​	​
0.48 (0.08,2.86)	0.91 (0.65,1.29)	CON	​	​
0.31 (0.04,2.30)	0.58 (0.05,7.37)	0.63 (0.05,7.88)	UFH + LDA	​
0.34 (0.18,0.67)	0.65 (0.12,3.51)	0.71 (0.14,3.71)	1.12 (0.17,7.57)	LDA

### 3.3 Risk of bias and publication bias

#### 3.3.1 Risk of bias by study design

Among the 25 RCTs included in the mixed-design analysis, the overall risk of bias was assessed as low in 20 studies, with 4 studies showing some concerns and 1 study identified as having a high risk of bias. In terms of randomization, 23 studies exhibited low risk, while 1 study was rated with some concerns, and 1 study was deemed to have a high risk of bias. Regarding deviations from the intended interventions, all 25 studies had a low risk of bias. With respect to missing outcome data, 24 studies demonstrated a low risk of bias, while 1 study had a high risk. For outcome measurement, 23 studies were considered to have low bias, and 2 studies were assessed as having some issues. In terms of selective reporting of results, all 25 studies were rated as having a low risk of bias (Supplementary Material 3).

For another studies, the NOS was employed to assess methodological quality. 3 studies received a score of 8, and 5 studies were scored 7 (revised from original 7 studies to align with 33 total included studies). All studies received the maximum score for “group selection” and “outcome assessment.” Among the 8 studies, 3 studies scored 2 points for the “comparability of groups” domain, while the remaining 5 studies scored 1 point.

#### 3.3.2 Publication bias

Publication bias was assessed using funnel plots (Supplementary Material 4) stratified by study design to identify design-specific publication bias. The scatter plots around the vertical axis exhibited varying degrees of symmetry, suggesting the potential for mild publication bias, with no meaningful differences in funnel plot symmetry between RCT and observational study designs. Specifically, Figure 3 show a relatively uniform distribution of points (no significant bias), whereas funnel plots in Figure 3 display some degree of asymmetry (possible publication bias) for both design types. The results of Egger’s test further supported these observations, with a p-value <0.05 for pre-eclampsia (suggesting potential bias for this outcome across all study designs), and p-values >0.05 for all remaining outcomes—indicating no clear evidence of design-specific or overall publication bias in the analysis. It should be acknowledged that funnel plot visualization and Egger-based statistical inference have limited reliability for publication bias assessment in the sparse networks and outcomes of this network meta-analysis, and the corresponding results need to be interpreted with caution.

## 4 Discussion

This comprehensive network meta-analysis included 33 studies, synthesize evidence from both RCTs and observational cohort studies, involving 5,858 singleton pregnancies at high risk for FGR. The study systematically evaluated the effects of various pharmacological treatments on both fetal and neonatal outcomes as well as pregnancy and delivery outcomes. Several key findings emerged from this analysis (Melamed et al., 2021): Both LMWH and the combination of LMWH + LDA demonstrated significant efficacy in reducing the incidence of IUGR, indicating their potential in improving fetal growth (Lees et al., 2022). LMWH treatment was found to significantly reduce the risk of several pregnancy complications, including placental abruption, preeclampsia, preterm birth, miscarriage, and cesarean section, highlighting its promise as a therapeutic choice for managing FGR-related complications (Figueras and Gratacós, 2014). LMWH + LDA exhibited particularly strong performance in preventing preeclampsia and increasing live birth rates, underscoring the additional benefits of this combination therapy in managing high-risk pregnancies. These findings are of great clinical importance, as they suggest that these pharmacological interventions can significantly improve both maternal and fetal health outcomes, offering valuable insights into the management of FGR and related complications in high-risk pregnancies.

IUGR is a critical indicator of fetal health and a common complication of FGR. IUGR is associated with an increased risk of stillbirth, preterm birth, neonatal morbidity, and long-term developmental challenges, making it a central focus in the management of high-risk pregnancies (Pels et al., 2020). This study found that both LMWH alone (OR = 0.40, 95% CI = 0.26, 0.62) and the combination of LMWH + LDA (OR = 0.37, 95% CI = 0.15, 0.93) significantly reduced the incidence of IUGR compared to the control group and LDA alone. These findings are consistent with previous research, which has shown that LMWH, particularly when combined with LDA, provides significant benefits in reducing the risk of IUGR in high-risk pregnancies (Cruz-Lemini et al., 2022). LDA in preventing IUGR likely involve multiple factors related to placental perfusion and thrombosis prevention. LMWH, an anticoagulant, improves uteroplacental blood flow by preventing the formation of thrombi in the placental vasculature. This can result in enhanced oxygen and nutrient delivery to the fetus, thus reducing the risk of restricted growth (González et al., 2025). LDA, an antiplatelet agent, contributes to this effect by further improving uteroplacental blood flow through its action on platelet aggregation and endothelial function. Together, LMWH + LDA work synergistically to reduce thrombotic events, enhance placental circulation, and prevent the complications associated with FGR and IUGR (Cruz-Lemini et al., 2022).

FGR is often associated with several pregnancy-related complications, which can adversely affect both maternal and fetal outcomes. Common complications in FGR pregnancies include placental abruption, preeclampsia, preterm birth, miscarriage, and cesarean delivery. These complications not only contribute to the high morbidity and mortality associated with FGR but also complicate management during pregnancy. In this study, five common pregnancy complications were included, and the results demonstrated that LMWH treatment significantly reduced the risk of several complications, including placental abruption (OR = 0.37, 95% CI = 0.14, 0.93), preeclampsia (OR = 0.21, 95% CI = 0.05, 0.93), preterm birth (OR = 0.61, 95% CI = 0.45, 0.81), miscarriage (OR = 0.42, 95% CI = 0.19, 0.91), and cesarean section (OR = 0.34, 95% CI = 0.18, 0.67). These findings highlight the therapeutic potential of LMWH in reducing IUGR and preeclampsia, as evidenced by high SUCRA rankings. However, while SUCRA values provide a probabilistic assessment of relative efficacy, their interpretation requires caution due to the absence of statistical significance for certain outcomes, such as birth weight. For instance, although LMWH and LMWH + LDA ranked highly in SUCRA analyses (e.g., Figure 3 for birth weight), the wide confidence intervals in pairwise comparisons (Table 1) indicate imprecision and potential heterogeneity. This limitation underscores that SUCRA rankings should not be overinterpreted without supporting statistical evidence, and future studies should address these gaps through larger sample sizes. Nonetheless, these findings collectively support the clinical relevance of LMWH-based therapies, consistent with previous studies indicating LMWH’s beneficial effects on pregnancy complications in women at risk for FGR. For instance, LMWH has been shown to improve placental blood flow and prevent thrombotic events, thereby mitigating risks of conditions like placental abruption and preeclampsia, which are closely linked to poor placental perfusion (Singh et al., 2024; Bailly et al., 2019). Additionally, LMWH’s anticoagulant properties may reduce the incidence of preterm birth and miscarriage by preventing the formation of clots in the uteroplacental vasculature, which can hinder fetal development. Cesarean section rates were also significantly reduced in LMWH-treated pregnancies, which may be attributed to the improved management of pregnancy complications and the reduction in adverse fetal outcomes. The mechanisms through which LMWH improves pregnancy outcomes in FGR patients likely involve its dual action as an anticoagulant and its impact on inflammation and placental function. By enhancing blood flow to the placenta and preventing clot formation, LMWH ensures better oxygen and nutrient delivery to the fetus, thus reducing the incidence of IUGR and associated complications (Pa et al., 2003). Furthermore, LMWH may modulate inflammatory pathways involved in the pathogenesis of preeclampsia, leading to better control of hypertensive disorders during pregnancy (Singh et al., 2024). Overall, these findings support the use of LMWH as an effective therapeutic strategy for managing FGR-related pregnancy complications and improving maternal and fetal health outcomes.

Preeclampsia and live birth rates are two critical outcomes for pregnancies affected by FGR. Preeclampsia, a hypertensive disorder of pregnancy, is often associated with poor placental perfusion and an increased risk of adverse maternal and fetal outcomes, including preterm birth, fetal demise, and maternal morbidity. Live birth rates, on the other hand, directly reflect the success of the pregnancy and the health of both the mother and the infant. In this study, the combination of LMWH and LDA was found to significantly reduce the incidence of preeclampsia (OR = 0.22, 95% CI = 0.08, 0.59) and significantly increase live birth rates (OR = 7.08, 95% CI = 2.16, 23.22) compared to LDA alone. These results are consistent with previous studies that have demonstrated the combined benefits of LMWH and LDA in managing high-risk pregnancies (Scarrone et al., 2024). Research has shown that LMWH, as an anticoagulant, improves uteroplacental blood flow by preventing thrombosis, which can reduce the incidence of preeclampsia—a condition linked to impaired placental perfusion (Shan et al., 2024). Additionally, LDA, a commonly used antiplatelet agent, contributes to improving endothelial function and reducing inflammation, both of which are critical in preventing the development of preeclampsia. The combination of LMWH + LDA works synergistically to target multiple pathways involved in the pathogenesis of preeclampsia, thus improving pregnancy outcomes. The mechanisms underlying the observed benefits of LMWH + LDA in preventing preeclampsia and improving live birth rates are multifaceted. First, LMWH’s ability to enhance placental blood flow through its anticoagulant effect reduces the risk of placental dysfunction and the development of hypertensive disorders such as preeclampsia. By preventing thrombotic events and ensuring better placental oxygenation, LMWH helps to maintain a healthier intrauterine environment, which may improve fetal growth and viability (Lin et al., 2022). Second, LDA’s role in reducing platelet aggregation and modulating inflammatory responses further enhances uteroplacental circulation, preventing the pathological changes associated with preeclampsia (Rolnik et al., 2022). Together, LMWH and LDA form a complementary treatment approach that targets both thrombotic and inflammatory processes, providing a robust strategy for improving maternal and fetal health outcomes in high-risk pregnancies.

LDA is commonly used in the management of FGR due to its antiplatelet properties, which are thought to improve uteroplacental blood flow and reduce the risk of thrombotic events that may contribute to fetal growth impairment. However, in this study, LDA alone ranked the lowest across multiple clinical outcomes, including the reduction of IUGR and the prevention of preeclampsia and miscarriage. These findings suggest that while LDA may have some benefits, its effectiveness is limited when used as a monotherapy in managing FGR. One potential explanation for LDA’s suboptimal performance in this analysis is its single mechanism of action. LDA primarily works by inhibiting platelet aggregation, thereby improving endothelial function and reducing inflammation. While this may help in improving placental perfusion, LDA alone may not be sufficient to address the multifactorial pathophysiology of FGR. FGR is often associated with placental insufficiency, thrombotic events, and systemic inflammation, and these factors may require more comprehensive treatment strategies (Rolnik et al., 2022). For instance, the combination of LDA with LMWH not only reduces thrombosis but also improves blood flow by enhancing placental circulation, leading to more favorable outcomes. The lack of synergy when LDA is used alone may explain its lower effectiveness compared to combination therapies. Furthermore, it is possible that the variability in the dosage and treatment duration of LDA across the studies included in this analysis contributed to the suboptimal ranking. Different doses of LDA may have varying effects on pregnancy outcomes, and some studies may not have utilized the most effective dosage for optimizing placental blood flow. The absence of a standardized treatment regimen for LDA in FGR management complicates its effectiveness and may have contributed to the observed variation in results (Luo et al., 2025). In contrast, combination therapies, such as LMWH + LDA, offer a more comprehensive approach by targeting multiple mechanisms involved in FGR and its associated complications, which could explain the superior outcomes observed in this study.

This study presents several notable strengths that contribute to the robustness of the findings. First, the use of a NMA allowed for the simultaneous comparison of multiple pharmacological interventions for FGR, including both those that have been directly compared in studies and those that have not. This comprehensive approach provides a more nuanced understanding of the comparative effectiveness of various treatments, offering insights that would be impossible to obtain from traditional pairwise meta-analysis. Second, this analysis synthesized data from a substantial number of studies, providing a large, diverse dataset that enhances the generalizability of the findings. This extensive evidence base strengthens the conclusions drawn from the analysis and provides a comprehensive assessment of the therapeutic options available for managing FGR. However, several limitations must also be considered when interpreting the results. First, despite the large number of studies included, the clinical heterogeneity among the studies was high, which may have affected the generalizability of the findings. Differences in patient populations, such as varying inclusion criteria and maternal comorbidities, as well as variations in treatment protocols (e.g., dosage and duration of therapy), could have introduced biases that influence treatment outcomes. We initially planned to control for partial heterogeneity through stratified analyses; however, the extremely small sample size of subgroup data after stratification precluded valid statistical analysis, leading to the abandonment of this approach, the potential for residual confounding remains. This heterogeneity underscores the need for more standardized clinical protocols and larger, well-controlled trials to confirm the efficacy of pharmacological interventions in FGR. Second, while the studies included in this meta-analysis were predominantly RCTs, the inclusion of non-randomized studies and observational data introduces a potential risk of bias. While observational studies are valuable for examining real-world clinical outcomes, they are inherently subject to confounding factors that can distort the relationship between treatment and outcomes. The inclusion of these studies, though necessary for providing a more comprehensive view of the available evidence, may limit the internal validity of the findings. Future research should focus on conducting high-quality RCTs to provide more definitive evidence regarding the efficacy of these treatments. In addition, Absence of formal GRADE-for-NMA certainty-of-evidence assessment: A standardized GRADE-for-NMA framework was not used to evaluate the certainty of evidence for the network treatment effect estimates in this study. This omission limits the ability to stratify the strength of inference for comparative treatment claims, as the certainty of evidence (e.g., for risk of bias, inconsistency, imprecision, publication bias) across intervention comparisons was not formally graded and quantified. Finally, the lack of data on long-term maternal and fetal outcomes represents a significant gap in the current literature. While this study provides valuable insights into short-term pregnancy-related outcomes such as fetal growth and preeclampsia, the long-term effects of these treatments on maternal cardiovascular health, infant development, and potential lifelong complications remain unclear. Further research should aim to assess the long-term safety and efficacy of pharmacological interventions for FGR to provide a more complete understanding of their benefits and risks.

## 5 Conclusion

This mixed-design network meta-analysis provides a comprehensive evaluation of pharmacological treatments for FGR, synthesizing both RCT evidence for treatment efficacy and observational evidence for real-world effectiveness, and highlighting the significant benefits of LMWH and its combination with LDA in improving fetal outcomes—reducing the incidence of IUGR, preeclampsia, and other complications while enhancing live birth rates. These findings suggest that LMWH and LMWH + LDA may serve as promising therapeutic options for managing high-risk pregnancies with FGR, notably specifying that they are applicable to high-risk FGR populations (e.g., those with thrombophilia, prior adverse outcomes, or placental vascular dysfunction) and may not be generalisable to all pregnant individuals. Despite the strengths of this study, further high-quality randomized controlled trials are urgently needed to substantiate the efficacy of combination therapies like LMWH + LDA, standardize treatment protocols, validate benefits across diverse high-risk subgroups, and explore long-term maternal and fetal outcomes, which will help to further confirm and refine the clinical application value of these therapeutic strategies for the optimal management of FGR and its associated complications.
