# Supplementary material for: Comparative effectiveness of pharmacological treatments for fetal growth restriction: a network meta-analysis
Source: Front Pharmacol. 2026 Apr 15;17:1792975. doi: 10.3389/fphar.2026.1792975 (PMC13124704; doi:10.3389/fphar.2026.1792975)
Supplement: Supplementary file 1 [file Supplementaryfile1.docx]

**Supplementary**

Table of Contents:

**[Supplementary 1: Search Strategy 2](#_Toc159076731)**

[Supplementary 2: Characteristics of studies and subjects included in the review 4](#_Toc159076743)

[Supplementary 3: Risk of Bias 2](#_Toc159076747)3

[Supplementary 4: Publication bias 2](#_Toc159076747)5

# Supplementary 1: Search Strategy

***Search Strategy（PubMed）:***

((((((((('hydroxymethylglutaryl coenzyme a reductase inhibitor'/exp OR statin*:ti,ab OR 'hydroxymethylglutaryl coa') AND reductase AND inhibit*:ti,ab OR 'hmg coa') AND reductase AND inhibit*:ti,ab OR hmgcoa) AND reductase AND inhibit*:ti,ab OR hmg) AND coa AND reductase AND inhibit*:ti,ab OR 'nitric oxide donor'/exp OR nitric) AND oxid*:ti,ab OR oxid) AND donor*:ti,ab OR no) AND releasing AND drug*:ti,ab OR 'no releasing') AND drug*:ti,ab AND (('pregnancy'/exp OR pregnan*:ti,ab OR 'fetus'/exp OR fetus*:ti,ab OR foetus*:ti,ab OR foetal*ti,ab OR fetal*:ti,ab OR intrauterine*:ti,ab OR uterin*:ti,ab OR gestation*:ti,ab) AND (complication*:ti,ab OR disease*:ti,ab OR retardation*:ti,ab OR restriction*:ti,ab OR weight*:ti,ab OR growth*:ti,ab OR 'growth'/exp) OR 'pregnancy complication'/exp OR 'fetus disease'/exp OR 'fetus weight'/exp OR 'intrauterine growth retardation'/exp OR 'low birth weight'/exp) OR (((((((('hydrogen sulfide'/exp OR hydrogen) AND sulfid*:ti,ab OR hydrogen) AND sulphid**:ti,ab OR 'proton pump inhibitor'/exp OR proton) AND pump AND inhibit*:ti,ab OR ppi*:ti,ab OR 'antiulcer agent'/exp OR 'anti ulcer') AND agent*:ti,ab OR anti) AND ulcer AND agent*:ti,ab OR 'esomeprazole'/exp OR esomeprazol*:ti,ab OR 'omeprazole'/exp OR omeprazol*:ti,ab OR pantoprazol*:ti,ab OR lansoprazol*:ti,ab OR rabeprazol*:ti,ab OR 'melatonin'/exp OR melatonin*:ti,ab OR 'creatine'/exp OR creatin*:ti,ab OR 'cysteine'/exp OR cystein*:ti,ab OR acetylcystein*:ti,ab OR 'insulin like') AND growth AND factor*:ti,ab OR igf*:ti,ab OR somatomedin*:ti,ab OR 'somatomedin'/exp OR mecasermin*:ti,ab OR somatropin*:ti,ab OR somatrem*:ti,ab OR sermorelin*:ti,ab OR tesamorelin*:ti,ab) AND (('pregnancy'/exp OR pregnan*:ti,ab OR 'fetus'/exp OR fetus*:ti,ab OR foetus*:ti,ab OR fetal*ti,ab OR fetal*:ti,ab OR intrauterine*:ti,ab OR uterin*:ti,ab OR gestation*:ti,ab) AND (complication*:ti,ab OR disease*:ti,ab OR retardation*:ti,ab OR restriction*:ti,ab OR weight*:ti,ab OR growth*:ti,ab OR 'growth'/exp) OR 'pregnancy complication'/exp OR 'fetus disease'/exp OR 'fetus weight'/exp OR 'intrauterine growth retardation'/exp OR 'low birth weight'/exp)) OR ((((aspirin*:ti,ab OR 'acetylsalicylic acid'/exp OR 'acetylsalicylic acid' OR acetylsalicylic) AND acid*:ti,ab OR 'antithrombocytic agent'/exp OR 'antithrombocytic agent' OR antiplatelet*:ti,ab OR antiaggregant*:ti,ab OR 'aggregation'/exp OR aggregation) AND inhibit*:ti,ab OR ditazole*:ti,ab OR cloricromen*:ti,ab OR clopidogrel*:ti,ab OR ticlopidine*:ti,ab OR dipyridamole*:ti,ab OR carbasalate*:ti,ab OR epoprostenol*:ti,ab OR indobufen*:ti,ab OR iloprost*:ti,ab OR aloxiprin*:ti,ab OR eptifibatide*:ti,ab OR tirofiban*:ti,ab OR triflusal*:ti,ab OR beraprost*:ti,ab OR treprostinil*:ti,ab OR prasugrel*:ti,ab OR cilostazol*:ti,ab OR ticagrelor*:ti,ab OR cangrelor*:ti,ab OR vorapaxar*:ti,ab OR selexipag*:ti,ab OR 'heparin'/exp OR 'heparin' OR heparin*:ti,ab OR ((('phosphodiesterase inhibitor'/exp OR phosphodiesterase) AND inhibit*:ti,ab OR 'sildenafil'/exp OR sildenafil*:ti,ab OR viagra*:ti,ab OR 'tadalafil'/exp OR tadalafil*:ti,ab OR cialis*:ti,ab OR 'vardenafil'/exp OR vardenafil*:ti,ab OR amrinone*:ti,ab OR milrinone*:ti,ab OR enoximone*:ti,ab OR cilostazol*:ti,ab OR inamrinone*:ti,ab OR levitra*:ti,ab OR dapoxetine*:ti,ab OR 'dapoxetine'/exp OR 'finasteride'/exp OR finasteride*:ti,ab OR 'vasculotropin'/exp OR vascular) AND endothelial AND growth AND factor*:ti,ab) OR vegf*:ti,ab OR (('microrna'/exp OR microrna*:ti,ab OR mirna*:ti,ab) AND (therap*:ti,ab OR treatment*:ti,ab OR medication*:ti,ab OR agent*:ti,ab OR drug*:ti,ab) NOT (screening*:ti,ab OR diagnos*:ti,ab))) AND (('pregnancy'/exp OR pregnan*:ti,ab OR 'fetus'/exp OR fetus*:ti,ab OR foetus*:ti,ab OR fetal*ti,ab OR fetal*:ti,ab OR intrauterine*:ti,ab OR uterin*:ti,ab OR gestation*:ti,ab) AND (complication*:ti,ab OR disease*:ti,ab OR retardation*:ti,ab OR restriction*:ti,ab OR weight*:ti,ab OR growth*:ti,ab OR 'growth'/exp) OR 'pregnancy complication'/exp OR 'fetus disease'/exp OR 'fetus weight'/exp OR 'intrauterine growth retardation'/exp OR 'low birth weight'/exp))) NOT ((mouse OR mice OR rat OR rats OR experimental) AND model* OR animal* OR preclinical* OR screening* OR diagnosis* OR diagnostic) NOT ([conference review]/lim OR [review]/lim)

# Supplementary 2: Characteristics of studies and subjects included in the review

| **Study** | **Country/Region** | **Study Design** | **Subjects  (intervention/ control)** | **Mean age  (intervention/ control)** | **Previous Clinical and Obstetric History** | **Clinical Characteristics at Index Pregnancy** | **Intervention detail** | | | | **Treatment Duration** | **Outcomes** |
| --- | --- | --- | --- | --- | --- | --- | --- | --- | --- | --- | --- | --- |
|  |  |  |  |  |  |  | **Intervention group** | | **Control group** | |  |  |
| Mohamed, 2014 | Egypt | Prospective non-randomized trial | 70 (47/23) | 28.7±3.3 vs. 29±3.55 | APS patients with a history of recurrent pregnancy loss, including miscarriages, IUFD, and live births. | Meeting the clinical and laboratory diagnostic criteria for antiphospholipid syndrome. | LMWH + LDA | Low-dose aspirin (81 mg daily) plus enoxaparin (40 mg subcutaneously daily). | LDA | Low-dose aspirin (81 mg daily). | Until the 35th week of gestation for aspirin and the 37th week for enoxaparin | Live birth, Miscarriage, Preterm delivery, Pre-eclampsia, IUGR, Birth Weight |
| Rasmark Roepke, 2019 | Sweden | RCT | 87 (45/42) | 33.0 ± 4.9 vs. 32.8 ± 4.6 | Recurrent pregnancy loss (RPL) | All women were in early pregnancy, confirmed by ultrasound. | LMWH | Tinzaparin sodium (LMWH), 4500 IU daily by subcutaneous injection. | CON | No treatment | Until 37 weeks of gestation | Live birth, Miscarriage, Preterm delivery, Pre-eclampsia, Cesarean, Small for Gestational Age, Fetal Death, Placental Abruption, Birth Weight |
| Badawy, 2008 | Egypt | RCT | 340 (170/170) | 26.2 ± 2.6 vs. 28.7 ± 3.1 | Previous spontaneous abortions; History of complications | All participants had a history of recurrent first trimester spontaneous abortions (3 or more losses) of unknown etiology. | LMWH | Low-molecular weight heparin (LMWH) (enoxaparin sodium 20 mg, 0.2 mL, once daily subcutaneous injection) plus folic acid (0.5 mg daily). | CON | Folic acid (0.5 mg daily) | Until 34 weeks of gestation | Miscarriage, Cesarean, Small for Gestational Age, Fetal Death, Placental Abruption, Birth Weight |
| Beaufils, 1985 | France | RCT | 102 (52/50) | 28.17±4.8 vs. 27.94±4.7 | High-risk women with a history of pre-eclampsia or fetal growth retardation, and women with vascular risk factors. | Pregnant women in their first trimester, high risk of pre-eclampsia or fetal growth retardation based on obstetric history and vascular risk factors. | Dipyridamole + LDA | Dipyridamole (300 mg daily) and Aspirin (150 mg daily). | CON | No treatment | From 3 months of gestation until delivery | Miscarriage, Pre-eclampsia, Placental Abruption, Birth Weight |
| Cowchock, 1997 | United States | RCT | 19 (11/8) | NA | Women with persistently positive antiphospholipid antibodies, considered low risk with a history of 0-2 spontaneous abortions. | The study involved low-risk pregnant women with persistently positive antiphospholipid antibody tests. | LDA | Low-dose aspirin (80 mg daily). | CON | No treatment | From 3 months of gestation until delivery | Fetal Death |
| de Vries, 2012 | The Netherlands, Australia, Sweden | RCT | 139 (70/69) | 29.1 ± 4.7 vs. 29.2 ± 4.4 | History of early-onset hypertensive disorders (pre-eclampsia, HELLP syndrome, eclampsia) and/or small-for-gestational age (SGA) infants. | Pregnant women <12 weeks gestation. History of early-onset hypertensive disorders (HD) or SGA infants in prior pregnancies. | LMWH + LDA | Low-molecular-weight heparin (LMWH) (dalteparin, 5000 IU subcutaneously daily) with aspirin (80 mg daily). | LDA | Aspirin (80 mg daily) alone | LMWH and aspirin were started before 12 weeks gestation and continued until delivery, with postpartum LMWH for 6 weeks. | Pre-eclampsia, Small for Gestational Age, Birth Weight |
| Dugalic, 2019 | Serbia | Prospective analytical cohort study | 358 (221/137) | 33.67 ± 4.01 | Women with inherited thrombophilias (including Factor V Leiden, Prothrombin G20210, MTHFR, and PAI-1) and a history of adverse pregnancy outcomes (APOs), including miscarriages, preterm birth, preeclampsia, and placental abruption. | Participants were referred between 11 and 15 weeks of gestation and followed until delivery. | LMWH | Low-molecular-weight heparin (LMWH) prophylaxis. | CON | No treatment | From 11-15 weeks of gestation until delivery. | Miscarriage, Preterm delivery, IUGR, Cesarean, Birth Weight |
| Fouda, 2011 | Egypt | RCT | 60 (30/30) | 27.47 ± 3.20 vs. 28.57 ± 3.48 | History of 3 or more consecutive spontaneous abortions before 10 weeks of gestation. | All participants had positive antiphospholipid antibodies and a history of recurrent spontaneous abortion. | LMWH + LDA | Low-molecular-weight heparin (LMWH) (enoxaparin 40 mg subcutaneously, once daily) plus low-dose aspirin (LDA) (75 mg once daily). | UFH + LDA | Unfractionated heparin (UFH) (5000 units subcutaneously, twice daily) plus low-dose aspirin (LDA) (75 mg once daily). | From the positive pregnancy test until delivery. | Live birth, Miscarriage, Preterm delivery, Pre-eclampsia, IUGR |
| Gris, 2004 | France | RCT | 160 (80/80) | 26.5 ± 4.0 vs. 27.2 ± 3.8 | One unexplained fetal loss after the 10th week of gestation. | All women had one prior unexplained pregnancy loss after 10 weeks of gestation. | LMWH | Low-molecular-weight heparin (LMWH) (enoxaparin 40 mg subcutaneously, once daily) plus Folic acid 5 mg daily. | LDA | Low-dose aspirin (LDA) (100 mg daily) plus Folic acid 5 mg daily. | From the 8th week of gestation until delivery. | Live birth, Miscarriage, Small for Gestational Age, Birth Weight |
| Gris, 2011 | France | RCT | 224 (112/112) | 29.1 ± 7.5 vs. 29.0 ± 9.0 | Women with a history of severe pre-eclampsia (PE) during their first pregnancy, without fetal loss, and negative for antiphospholipid antibodies. | All women had severe pre-eclampsia during their first pregnancy, defined by hypertension and significant proteinuria after 20 weeks of gestation. | LMWH + LDA | Enoxaparin (40 mg) subcutaneously once daily, starting from the positive pregnancy test, combined with low-dose aspirin (100 mg) daily, Folic acid supplementation (5 mg daily) was provided to all women. | LDA | Low-dose aspirin (100 mg) daily, combined with folic acid. | From the positive pregnancy test until delivery, with enoxaparin continued postpartum for 6 weeks. | Pre-eclampsia, Cesarean, Placental Abruption |
| Groom, 2017 | The Netherlands, Australia | RCT | 160 (80/80) | NA | Women with a history of preeclampsia and/or small-for-gestational-age (SGA) babies, considered high-risk for recurrence of preeclampsia and FGR/IUGR in the subsequent pregnancy. | All women were in their second pregnancy, with a history of either preeclampsia or small-for-gestational-age pregnancies in the first pregnancy. | LMWH | Enoxaparin (40 mg) subcutaneously once daily, combined with standard high-risk care (such as folic acid, blood pressure monitoring, etc.). | CON | Standard care (blood pressure monitoring, folic acid, lifestyle modifications, etc.) without enoxaparin. | From the beginning of the second pregnancy until delivery. | Preterm delivery, Pre-eclampsia, IUGR, Small for Gestational Age, Birth Weight |
| Haddad, 2016 | Egypt | RCT | 100 (50/50) | 29.8 ± 3.2 vs. 30.5 ± 3.1 | Women with a history of severe preeclampsia before 34 weeks of gestation. | All participants were pregnant women with a history of severe preeclampsia and at high risk for preterm birth and complications like IUGR. | LMWH + LDA | Low-molecular-weight heparin (LMWH) (enoxaparin 40 mg subcutaneously, once daily) plus low-dose aspirin (LDA) (75 mg daily), combined Folic acid supplementation (5 mg daily) for all women. | LDA | Low-dose aspirin (75 mg daily) alone, without LMWH. Folic acid supplementation (5 mg daily) for all women. | From 12 weeks of gestation until delivery. | Preterm delivery, Pre-eclampsia, Small for Gestational Age, Fetal Death, Placental Abruption, Birth Weight |
| Talari, 2014 | Iran | RCT | 80 (40/40) | 27.8 ± 4.5 vs. 27.0 ± 5.9 | Women with high-risk factors for preeclampsia, including a history of preeclampsia, essential hypertension, positive family history, or underlying vascular disorder, and those with gestational diabetes, maternal age < 20 years or > 40 years. | All women underwent Doppler ultrasonography at 12-16 weeks of pregnancy to assess uterine artery flow. Those with abnormal findings (unilateral notch with RI ≥ 0.65 or bilateral notch with RI ≥ 0.55) were included in the study. | LDA | Low-dose aspirin 80 mg daily. | CON | Placebo | From 12-16 weeks of gestation until delivery. | Preterm delivery, Pre-eclampsia, IUGR, Birth Weight |
| Kayatas, 2014 | Turkey | Prospective observational study | 150 (100/50) | 28 ± 5 vs. 28.8 ± 6 | Women with a history of recurrent pregnancy loss (2 or more unexplained first trimester losses). | Women were in their first trimester, with unexplained recurrent miscarriage (URM). | LMWH | Low molecular weight heparin (LMWH): 4000 IU/day enoxaparin or 3500 IU/day tinzaparin subcutaneously. | CON | No thromboprophylaxis (no medication given). | From 6 weeks of gestation until delivery. | Live birth, Miscarriage, Preterm delivery, IUGR, Birth Weight |
| Kupferminc, 2011a | Israel | Retrospective study | 116 (87/29) | 32.19 ± 6.2 vs. 32.6 ± 5.0 | Women with inherited thrombophilia (e.g., factor V Leiden, prothrombin mutations, protein S or C deficiencies) and a history of severe pregnancy complications such as severe preeclampsia, fetal growth restriction (FGR <5th percentile), placental abruption, and stillbirth. | All women had a history of severe preeclampsia, FGR, placental abruption, or stillbirth during previous pregnancies. | LMWH | LMWH (Enoxaparin) 1 mg/kg from 5–15 weeks of pregnancy. | CON | No treatment (referred only for delivery). | From 5–15 weeks and continued throughout pregnancy. | Pre-eclampsia, IUGR, Placental Abruption, Birth Weight |
| Kupferminc, 2011b | Israel | Retrospective study | 72 (32/40) | 31.8 ± 5.2 vs. 31.9 ± 4.3 | Women with severe preeclampsia, FGR (less than 5th percentile), severe placental abruption, and stillbirth after 20 weeks of gestation, with a history of placental vasculopathy in a previous pregnancy. | All participants had a history of severe pregnancy complications such as FGR, placental abruption, and stillbirth in a previous pregnancy. | LMWH | Low molecular weight heparin (LMWH) (enoxaparin sodium 1 mg/kg, administered from 5–15 weeks of gestation until delivery). | CON | No treatment | From 5–15 weeks of pregnancy until delivery. | Pre-eclampsia, IUGR, Placental Abruption, Birth Weight |
| Kutteh, 1996 | United States | Prospective, single-center trial | 50 (25/25) | 33.2 ± 4.2 vs. 33.5 ± 5.8 | All participants had a history of at least three consecutive spontaneous miscarriages and positive antiphospholipid antibodies (APA) on at least two separate occasions. | The women had a mean of 4.6 pregnancies, with an average of 3.9 prior miscarriages and 0.7 prior live births. | UFH + LDA | Heparin (5000 units) twice daily and low-dose aspirin (81 mg) daily, initiated after confirmed pregnancy (mean of 5.3 weeks gestation). | LDA | Low-dose aspirin (81 mg) daily. | From 5.3 ± 1.1 weeks gestation until delivery. | Live birth, Miscarriage, Preterm delivery, Pre-eclampsia, IUGR, Cesarean, Birth Weight |
| Martinelli, 2012 | Italy | RCT | 135 (67/68) | 34±5.2 vs. 34±6.1 | Women with a history of severe pregnancy complications, including pre-eclampsia, HELLP syndrome, fetal intrauterine death, fetal growth restriction (FGR), placental abruption, and multiple early miscarriages. | All participants were considered high-risk pregnancies with a history of severe pregnancy complications including FGR, pre-eclampsia, placental abruption, and previous fetal loss. | LMWH | Nadroparin (3800 IU, subcutaneous injection daily) plus medical monitoring and care. | CON | Medical monitoring and care only. | April 2007 to April 2010. | Preterm delivery, IUGR, Cesarean, Fetal Death, Placental Abruption, Birth Weight |
| Mello, 2004 | Brazil | Non-randomized trial | 90 (45/45) | 29.8 ± 3.2 vs. 30.5 ± 3.1 | Women with a history of severe preeclampsia and recurrent pregnancy loss due to hypertension, who were at high risk for pregnancy complications including FGR. | Participants were pregnant women at high risk of recurrent preeclampsia and FGR, with a focus on preeclampsia recurrence in subsequent pregnancies. | LMWH | Low-molecular-weight heparin (LMWH) 5000 units daily (subcutaneously) from first trimester until delivery. | CON | No heparin treatment (only medical care). | From first trimester of pregnancy until delivery. | Miscarriage, Preterm delivery, Pre-eclampsia, IUGR, Cesarean, Fetal Death, Placental Abruption, Birth Weight |
| Odibo, 2015 | United States | RCT | 53 (30/23) | 31.6 ± 6.1 vs. 30.0 ± 5.0 | Chronic Hypertension: 10/14 (71.4%) in placebo group, 6/16 (37.5%) in aspirin group Pregestational Diabetes: 2/14 (14.3%) in placebo group, 4/16 (25.0%) in aspirin group | BMI: 36.6 ± 6.9 vs. 37.4 ± 8.9 (aspirin vs. placebo groups) Risk score: 5.9 ± 3.2 vs. 5.0 ± 2.9 (aspirin vs. placebo groups) | LDA | 80 mg/day of low-dose aspirin from 11+0 to 13+6 weeks until 37 weeks or delivery | CON | Placebo | From 11+0 to 13+6 weeks until 37 weeks or delivery | Pre-eclampsia, Small for Gestational Age |
| Pasquier, 2015 | France | RCT | 258 (138/120) | 32.7 ± 5.2 vs. 32.1 ± 5.4 | Previous Miscarriages: 100 (72.5%) in enoxaparin group, 86 (72.9%) in placebo group had 3 or more previous miscarriages | BMI: 23.9 ± 4.4 vs. 23.9 ± 5.0 (enoxaparin vs. placebo group) Systolic Blood Pressure: 118 ± 11 vs. 118 ± 12 (enoxaparin vs. placebo group) Diastolic Blood Pressure: 69 ± 9 vs. 67 ± 9 (enoxaparin vs. placebo group) Gestation at Inclusion: 39.1 ± 10.3 days vs. 38.9 ± 9.3 days (enoxaparin vs. placebo group) | LMWH | Enoxaparin 40 mg daily via subcutaneous injection until 35 weeks’ gestation | CON | Placebo (saline solution) via subcutaneous injection until 35 weeks’ gestation | From enrollment (ideally before 5 weeks’ gestation) to 35 weeks’ gestation | Live birth, Miscarriage, Preterm delivery, Pre-eclampsia, Small for Gestational Age |
| Schleussner, 2015 | Germany, Austria | RCT | 449 (226/223) | 31.9 ± 5.0 vs. 32.3 ± 5.3 | Previous Miscarriages: 2.6 ± 1.4 vs. 2.6 ± 1.4 (LMWH vs. control group) | Gestational Age at Inclusion: 5-8 weeks of gestation  BMI: 23.9 ± 5.2 vs. 24.1 ± 5.3 (LMWH vs. control group)  Systolic BP: 116.2 ± 11.3 vs. 115.5 ± 10.8 (LMWH vs. control group)  Diastolic BP: 75.2 ± 8.4 vs. 74.8 ± 8.1 (LMWH vs. control group) | LMWH | Dalteparin-sodium (5000 IU daily) + multivitamins | CON | Multivitamins only | Until 24 weeks of gestation | Live birth, Preterm delivery, Pre-eclampsia, Small for Gestational Age, Placental Abruption |
| Sergio, 2006 | Italy | Non-randomized trial | 54 (23/31) | 29 ± 4.2 vs. 28.7 ± 4.8 | Previous Severe Preeclampsia: 100% in both groups Previous IUGR/Low Birth Weight: 100% in both groups | Gestational Age at Enrollment: 6-10 weeks gestation BMI: 24.8 ± 3.5 vs. 24.1 ± 3.9 (LMWH + LDA vs. LDA group) Systolic BP: 112 ± 5 vs. 110 ± 6 (LMWH + LDA vs. LDA group) Diastolic BP: 69 ± 5 vs. 70 ± 6 (LMWH + LDA vs. LDA group) | LMWH + LDA | Enoxaparin 4000 IU daily subcutaneously + low-dose aspirin (100 mg/day) | LDA | Low-dose aspirin (100 mg/day) | From positive pregnancy test to delivery | Preterm delivery, Small for Gestational Age, Fetal Death, Birth Weight |
| Shaaban, 2016 | Egypt | RCT | 300 (150/150) | 26.61 ± 3.23 vs. 26.63 ± 3.64 | Recurrent Miscarriage (≥3 miscarriages): 100% in both groups | Gestational Age at Enrollment: Positive pregnancy test, between 4 and 8 weeks BMI: 25.4 ± 2.5 vs. 25.6 ± 2.3 (LMWH vs. control group) | LMWH | LMWH (Tinzaparin sodium 4500 IU/day) + Folic acid (500 mg/day) | CON | Folic acid only (500 mg/day) | Daily subcutaneous injections from positive pregnancy test until the 20th week of gestation | Miscarriage, Preterm delivery |
| Tulppala, 1997 | Finland | RCT | 66 (33/33) | NA | Recurrent spontaneous abortion (RSA), with or without detectable ACA | Participants had RSA and either elevated or normal ACA levels | LDA | Low-dose aspirin (50 mg/day) from detection of pregnancy until delivery | CON | Placebo (similar-looking pill) | From pregnancy test confirmation to delivery | Miscarriage, Pre-eclampsia, IUGR, Cesarean, Birth Weight |
| Uzan, 1991 | France | RCT | 229 (156/73) | 30.4 ± 4.5 vs. 29.6 ± 4.4 | Patients had one or two previous poor pregnancy outcomes, at least one of which involved fetal growth retardation. | Pregnant women at 15-18 weeks gestation, with history of poor pregnancy outcomes (e.g., fetal growth retardation, fetal death, abruptio placentae). | LDA | Low-dose aspirin (150 mg/day) | Dipyridamole + LDA | Aspirin plus dipyridamole (150 mg/day + 225 mg/day) | Throughout pregnancy, starting from 15-18 weeks of gestation | IUGR, Fetal Death, Placental Abruption, Birth Weight |
| Van Hoorn, 2016 | The Netherlands, Australia, Sweden | RCT | 32 (16/16) | 33.6 ± 5.3 vs. 30.3 ± 4.2 | Pre-eclampsia, HELLP syndrome, or eclampsia | Gestational age at entry: 59.9 ± 15.3 vs. 65.9 ± 13.4 days Chronic hypertension: 4/16 (25%) vs. 4/16 (25%) Pregnancy interval: 1088 ± 969.6 days vs. 553 ± 327.9 days Body Mass Index (BMI): 26.7 ± 6.4 vs. 26.3 ± 7.6 kg/m² | LMWH + LDA | Dalteparin 5000 IU daily with aspirin 80 mg daily | LDA | Aspirin 80 mg daily only | From 6–12 weeks gestation until delivery | Miscarriage, Preterm delivery, Pre-eclampsia, Small for Gestational Age, Birth Weight |
| Furuhashi, 2019 | Japan | RCT | 26 (12/14) | 37.5 ±3.6 vs. 38.0 ±3.3 | Gestational hypertension, preeclampsia, superimposed preeclampsia No chronic hypertension reported | Gestational week at registration: 28.6 (26.1–31.4) weeks (tadalafil group), 29.0 (25.3–30.7) weeks (conventional group) Maternal BMI: 25.0 (22.5–26.4) kg/m² (tadalafil group), 26.7 (24.7–30.5) kg/m² (conventional group) | Tadalafil | Tadalafil treatment group: 20 mg oral tadalafil daily | CON | Standard management of HDP | From randomization until delivery | IUGR, Birth Weight |
| Mirzamoradi, 2023 | Iran | RCT | 107 (54/53) | 31 ± 5 vs. 30 ± 5 | History of spontaneous PTD: 89% vs. 79% | Gravidity: 39% (2) vs. 44% (2), 46% (3-4) vs. 44% (3-4), 15% (≥5) vs. 12% (≥5) Abortion history: None (63% vs. 64%), 1 (17% vs. 23%), 2 (13% vs. 9%), ≥3 (7% vs. 4%) | LDA | Low-dose aspirin (80 mg daily until 36 weeks) plus classic treatment | CON | Classic treatment only | Until 36 weeks of pregnancy | Preterm delivery |
| Abdi, 2020 | Iran | RCT | 90 (45/45) | 29.5 ± 5.5 vs. 31.1 ± 6.4 | Women with a history of preeclampsia in a previous pregnancy | Singleton pregnancy, gestational age 12-15 weeks at enrollment, with no chronic medical diseases, abnormal uterine artery Doppler, or abnormal PAPP-A values. | LDA | 80 mg of aspirin daily | CON | Placebo | From 12-15 weeks of gestation until the 36th week of pregnancy | Preterm delivery, Pre-eclampsia, IUGR, Birth Weight |
| Chen, 2023 | China | RCT | 898 (464/434) | NA | High-risk factors for pre-eclampsia, including history of pre-eclampsia, chronic hypertension, or diabetes mellitus, obesity, advanced maternal age, family history of pre-eclampsia, and nulliparity | Singleton pregnancy, gestational age between 12-20 weeks at enrollment | LDA | 100 mg of low-dose aspirin daily from recruitment until 34 weeks or early delivery | CON | Standard antenatal care without aspirin | 12–34 weeks of gestation | Pre-eclampsia |
| Huai, 2021 | China | RCT | 397 (137/260) | 32.4 ± 4.7 vs. 32.1 ± 4.2 | High-risk for preeclampsia, with factors including previous preeclampsia, obesity (BMI ≥ 28), advanced maternal age (≥35), family history of preeclampsia, and nulliparity. | Singleton pregnancy, gestational age between 12 and 20 weeks, stage 1 hypertension (systolic BP 130–139 mmHg or diastolic BP 80–89 mmHg) as per the updated ACC/AHA guidelines, and normotension. | LDA | 100 mg/day of enteric-coated aspirin from 12–20 weeks until 34 weeks of gestation. | CON | Standard antenatal care without aspirin. | 12–34 weeks of gestation. | Preterm delivery, Pre-eclampsia, Placental Abruption, Birth Weight |
| Landman, 2022 | The Netherlands | RCT | 387 (194/193) | 32.5 ± 3.8 | Previous spontaneous preterm birth, a history of cervical surgery, uterine surgery, or multiple preterm births. | Singleton pregnancy, randomization between 8 and 16 weeks, previous spontaneous preterm birth between 22 and 37 weeks. | LDA | 80 mg daily low-dose aspirin from 8–16 weeks until 36 weeks of gestation or delivery. | CON | Placebo | 8–16 weeks to 36 weeks of gestation or delivery. | Preterm delivery, Small for Gestational Age, Placental Abruption, Birth Weight |

Note: CON, Control Group; LDA, low-dose aspirin; LMWH, low molecular weight heparin; UFH, unfractionated heparin; IUGR, Intrauterine Growth Restriction.

# Supplementary 3: Risk of Bias

## Table 3.1 The Rob.2 Scale for included studies

| **Author** | **Bias arising from the randomization process** | **Bias due to deviations from intended intervention** | **Bias due to missing outcome data** | **Bias in measurement of the outcome** | **Bias in selection of the reported result** | **Overall** |
| --- | --- | --- | --- | --- | --- | --- |
| Rasmark Roepke, 2019 | Low | Low | Low | Low | Low | Low |
| Badawy, 2008 | Low | Low | Low | Low | Low | Low |
| Beaufils, 1985 | Low | Low | Low | Low | Low | Low |
| Cowchock, 1997 | High | Low | High | Low | Low | High |
| de Vries, 2012 | Low | Low | Low | Low | Low | Low |
| Fouda, 2011 | Low | Low | Low | Low | Low | Low |
| Gris, 2004 | Low | Low | Low | Some concerns | Low | Some concerns |
| Gris, 2011 | Low | Low | Low | Low | Low | Low |
| Groom, 2017 | Low | Low | Low | Low | Low | Low |
| Haddad, 2016 | Low | Low | Low | Low | Low | Low |
| Talari, 2014 | Low | Low | Low | Some concerns | Low | Some concerns |
| Martinelli, 2012 | Low | Low | Low | Low | Low | Some concerns |
| Odibo, 2015 | Low | Low | Low | Low | Low | Low |
| Pasquier, 2015 | Low | Low | Low | Low | Low | Low |
| Schleussner, 2015 | Low | Low | Low | Low | Low | Low |
| Shaaban, 2016 | Low | Low | Low | Low | Low | Low |
| Tulppala, 1997 | Low | Low | Low | Low | Low | Low |
| Uzan, 1991 | Low | Low | Low | Low | Low | Low |
| Van Hoorn, 2016 | Low | Low | Low | Low | Low | Low |
| Furuhashi, 2019 | Low | Low | Low | Low | Low | Low |
| Mirzamoradi, 2023 | Low | Low | Low | Low | Low | Low |
| Abdi, 2020 | Some concerns | Low | Low | Low | Low | Some concerns |
| Chen, 2023 | Low | Low | Low | Low | Low | Low |
| Huai, 2021 | Low | Low | Low | Low | Low | Low |
| Landman, 2022 | Low | Low | Low | Low | Low | Low |

## Table 3.2 The Newcastle-Ottawa Quality Assessment Scale for included controlled studies

| **Study** | Selection of the study groups | Comparability of the groups | Outcome | Total score |
| --- | --- | --- | --- | --- |
| Mohamed, 2014 | ☒☒☒☒ | ☒ | ☒☒ | 7 |
| Dugalic, 2019 | ☒☒☒☒ | ☒☒ | ☒☒ | 8 |
| Kayatas, 2014 | ☒☒☒☒ | ☒ | ☒☒ | 7 |
| Kupferminc, 2011a | ☒☒☒☒ | ☒☒ | ☒☒ | 8 |
| Kupferminc, 2011b | ☒☒☒☒ | ☒☒ | ☒☒ | 8 |
| Kutteh, 1996 | ☒☒☒☒ | ☒ | ☒☒ | 7 |
| Mello, 2004 | ☒☒☒☒ | ☒ | ☒☒ | 7 |

# Supplementary 4: Publication bias


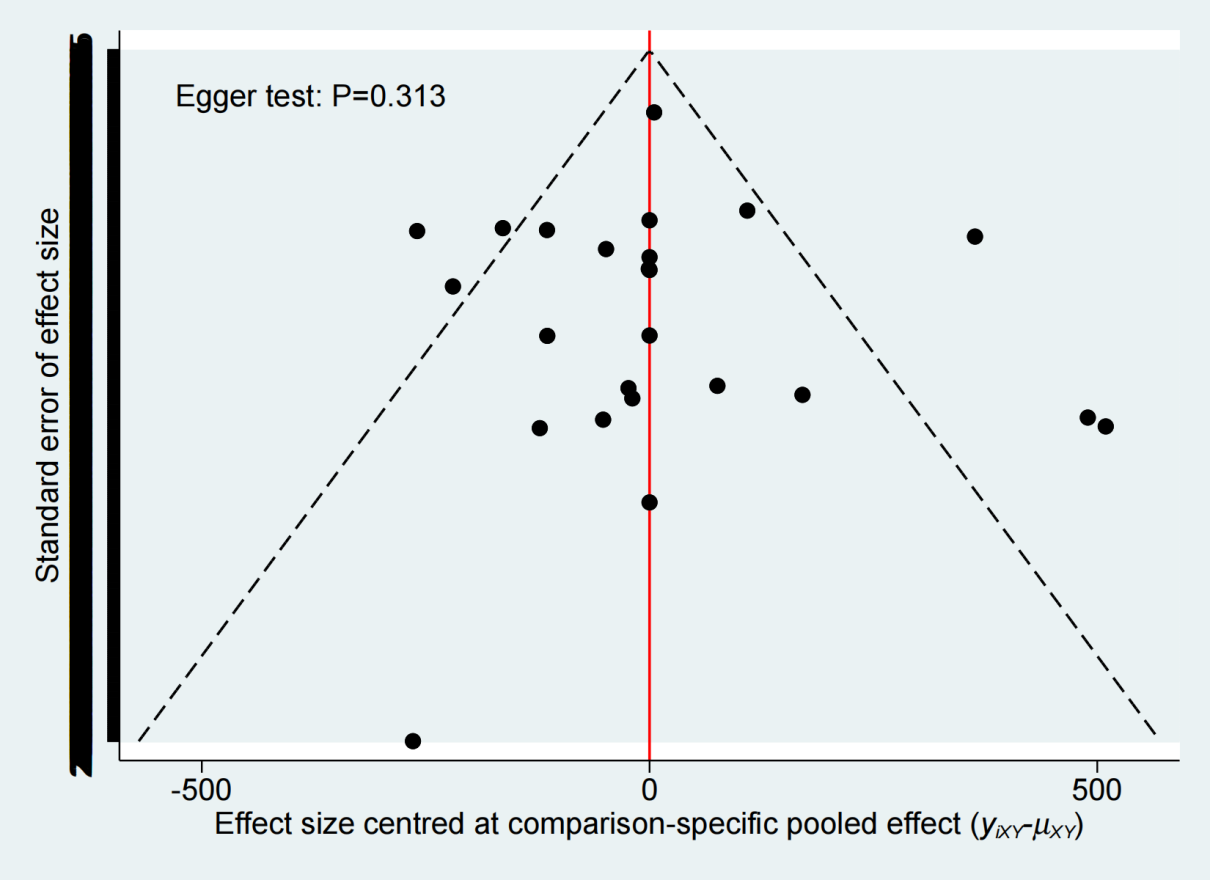


Figure 4.1 The funnel plot of Birth Weight. The result of Egger test showed the p=0.313.


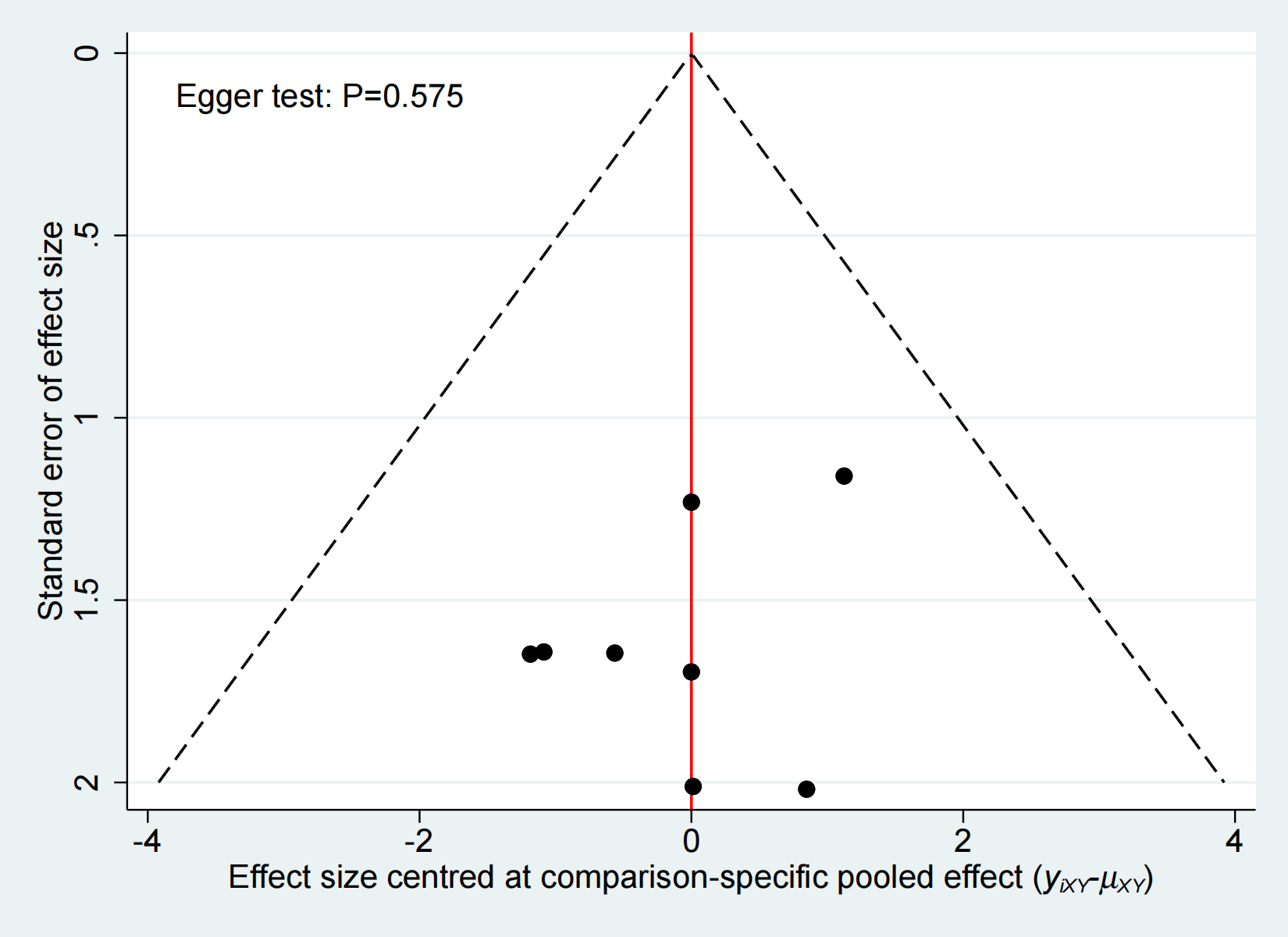


Figure 4.2 The funnel plot of Fetal Death. The result of Egger test showed the p=0.575.


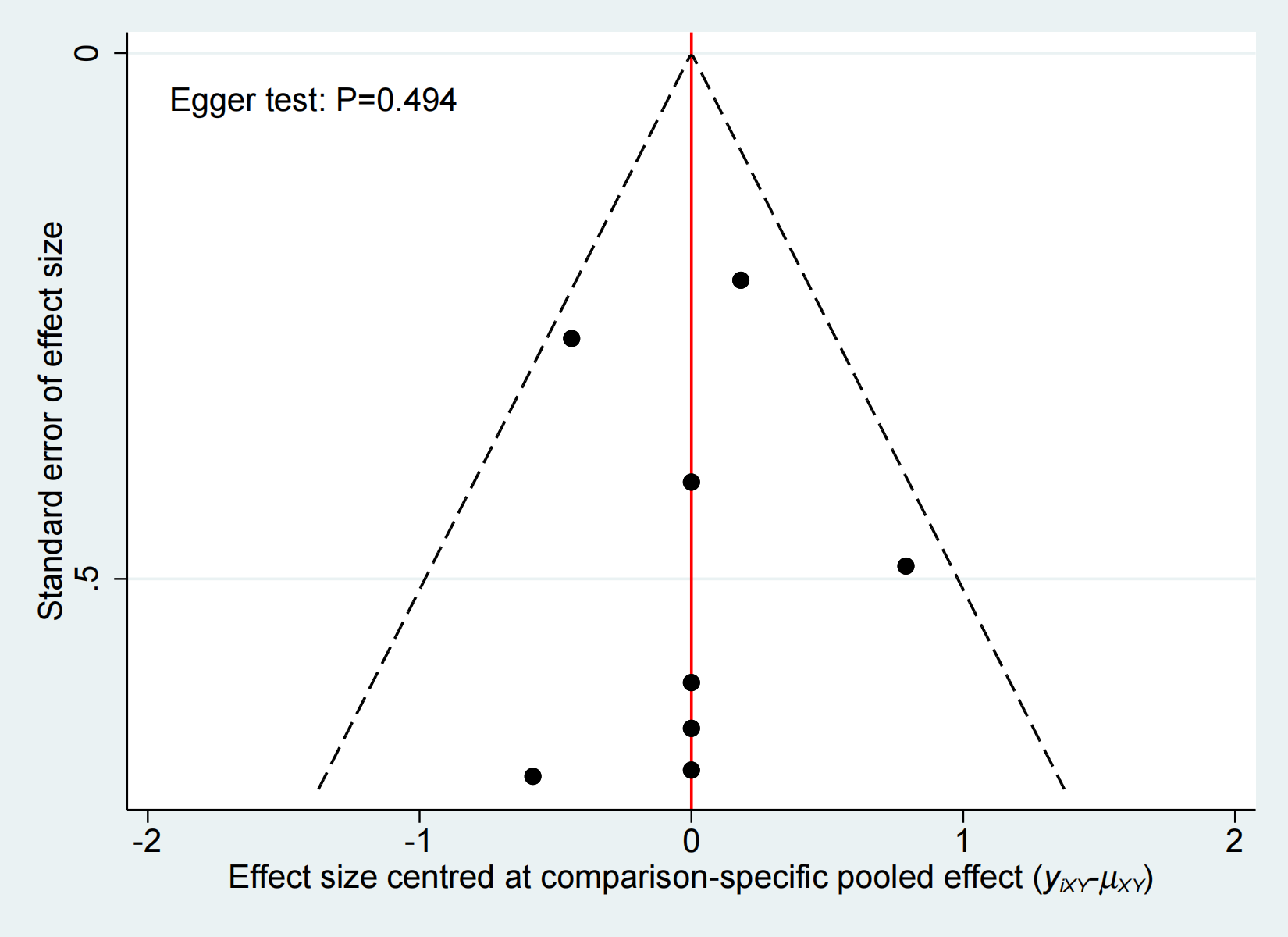


Figure 4.3 The funnel plot of Live Birth. The result of Egger test showed the p=0.494.


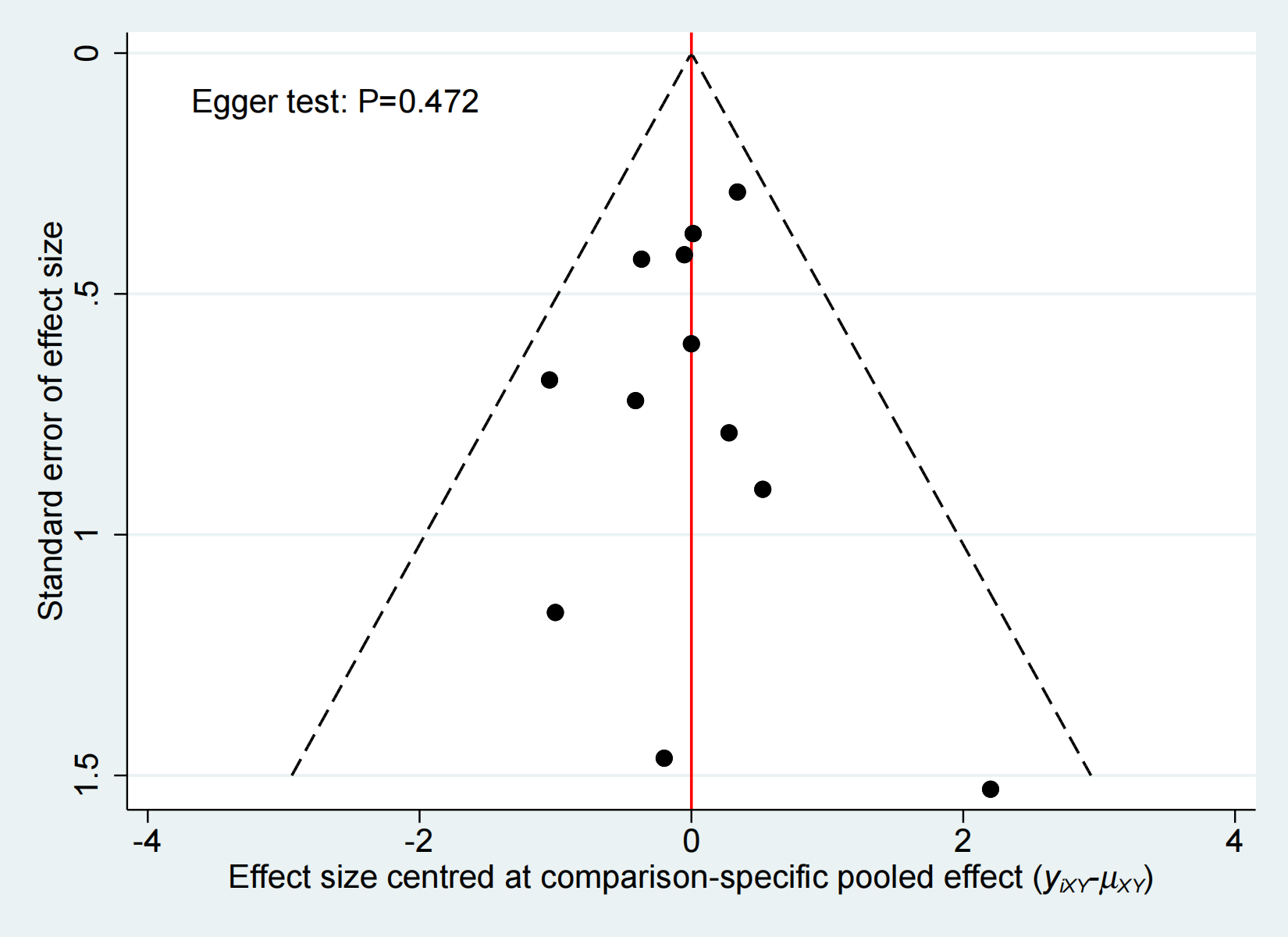


Figure 4.4 The funnel plot of Small for Gestational Age. The result of Egger test showed the p=0.472.


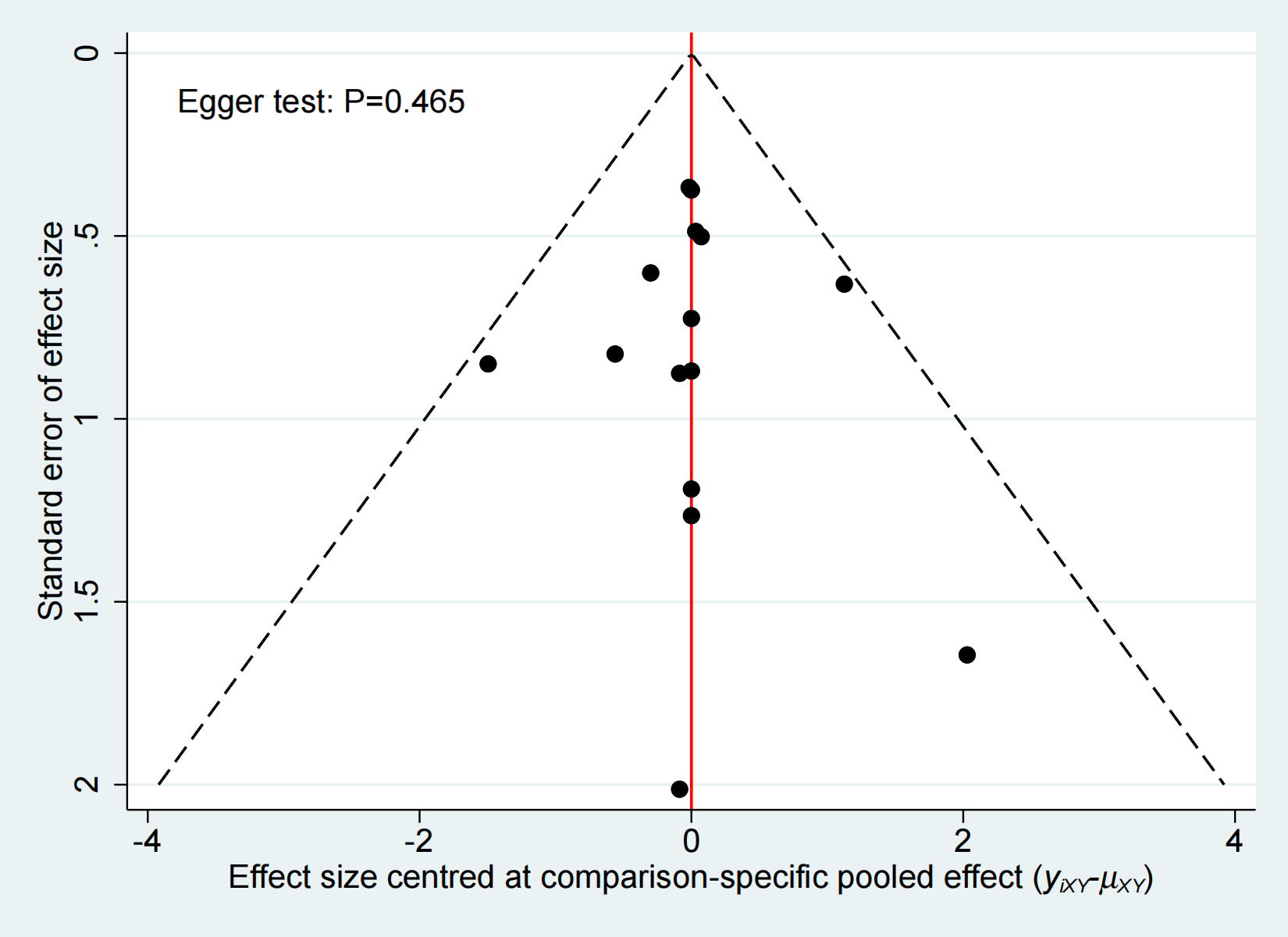


Figure 4.5 The funnel plot of IUGR. The result of Egger test showed the p=0.465.


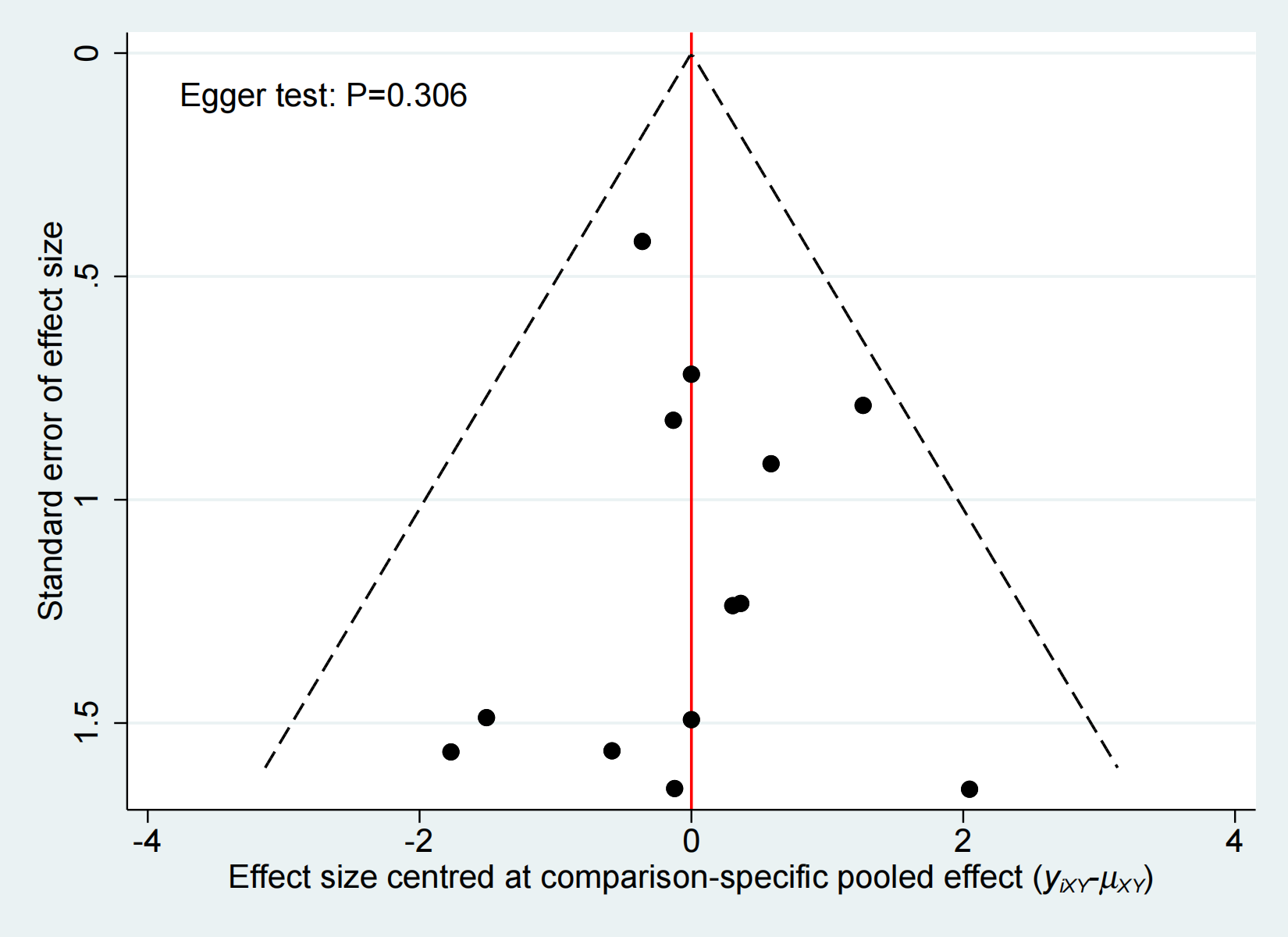


Figure 4.6 The funnel plot of Placental Abruption. The result of Egger test showed the p=0.306.


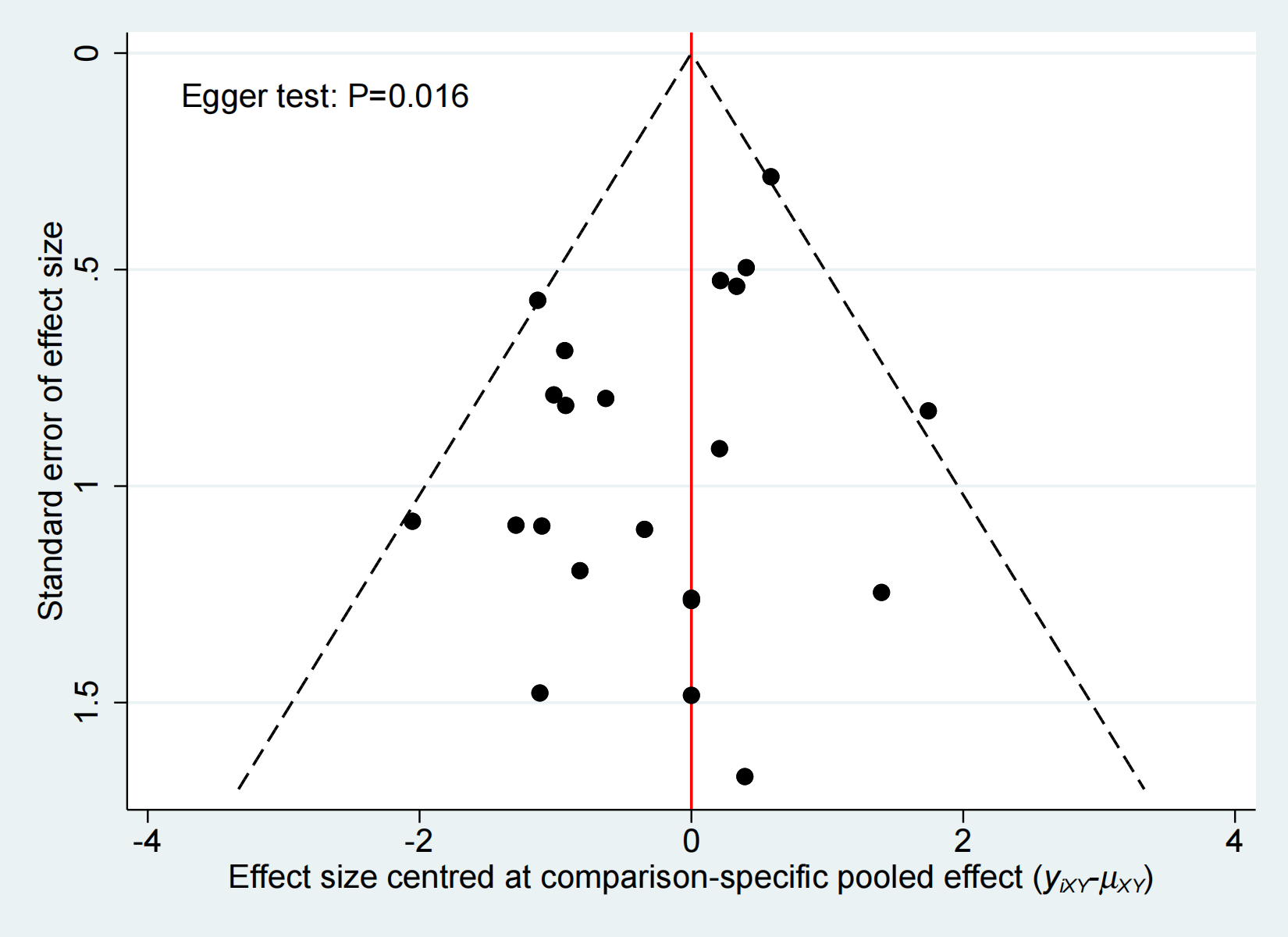


Figure 4.7 The funnel plot of Pre-eclampsia. The result of Egger test showed the p=0.016.


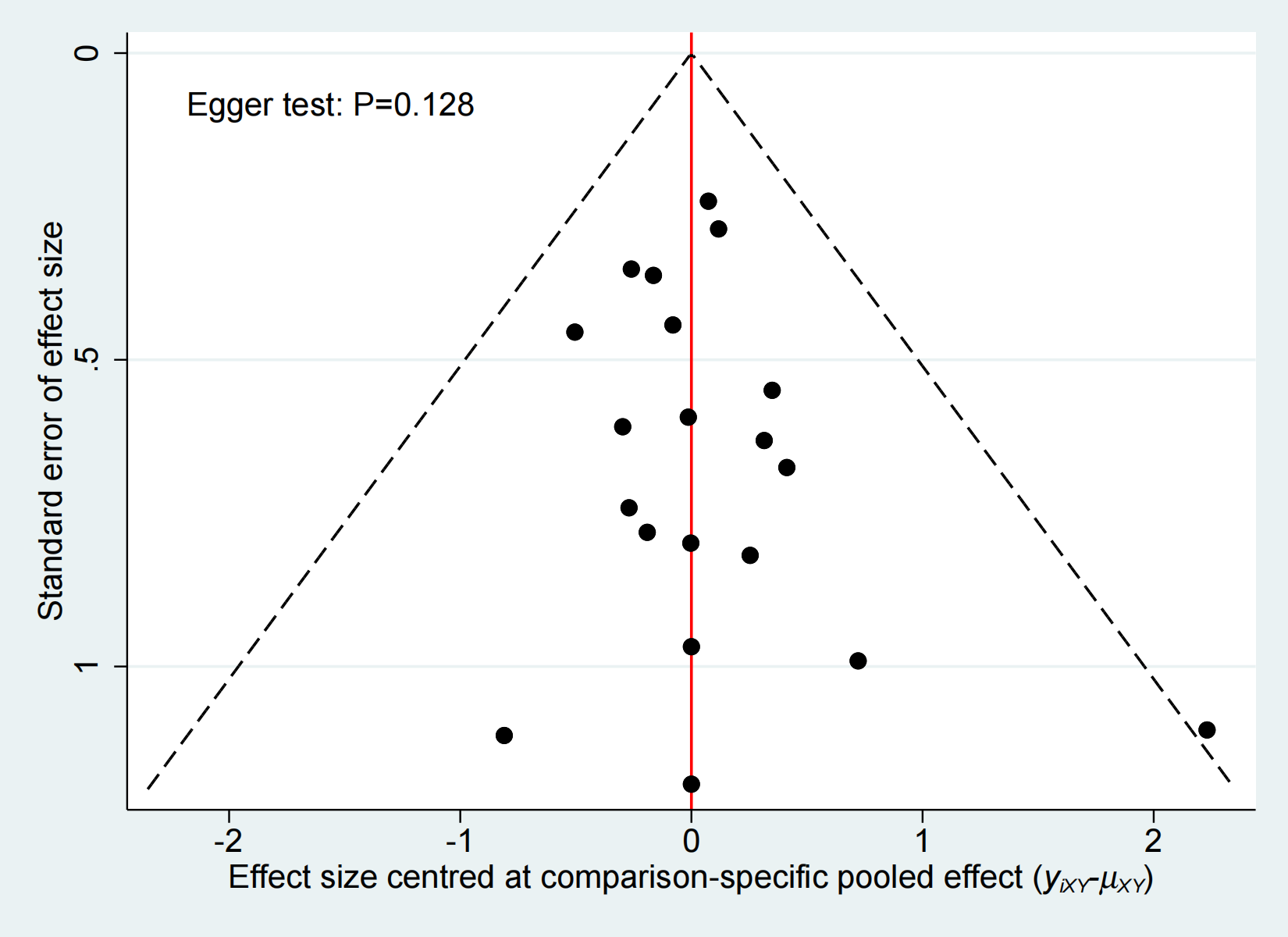


Figure 4.8 The funnel plot of Preterm Delivery. The result of Egger test showed the p=0.128.


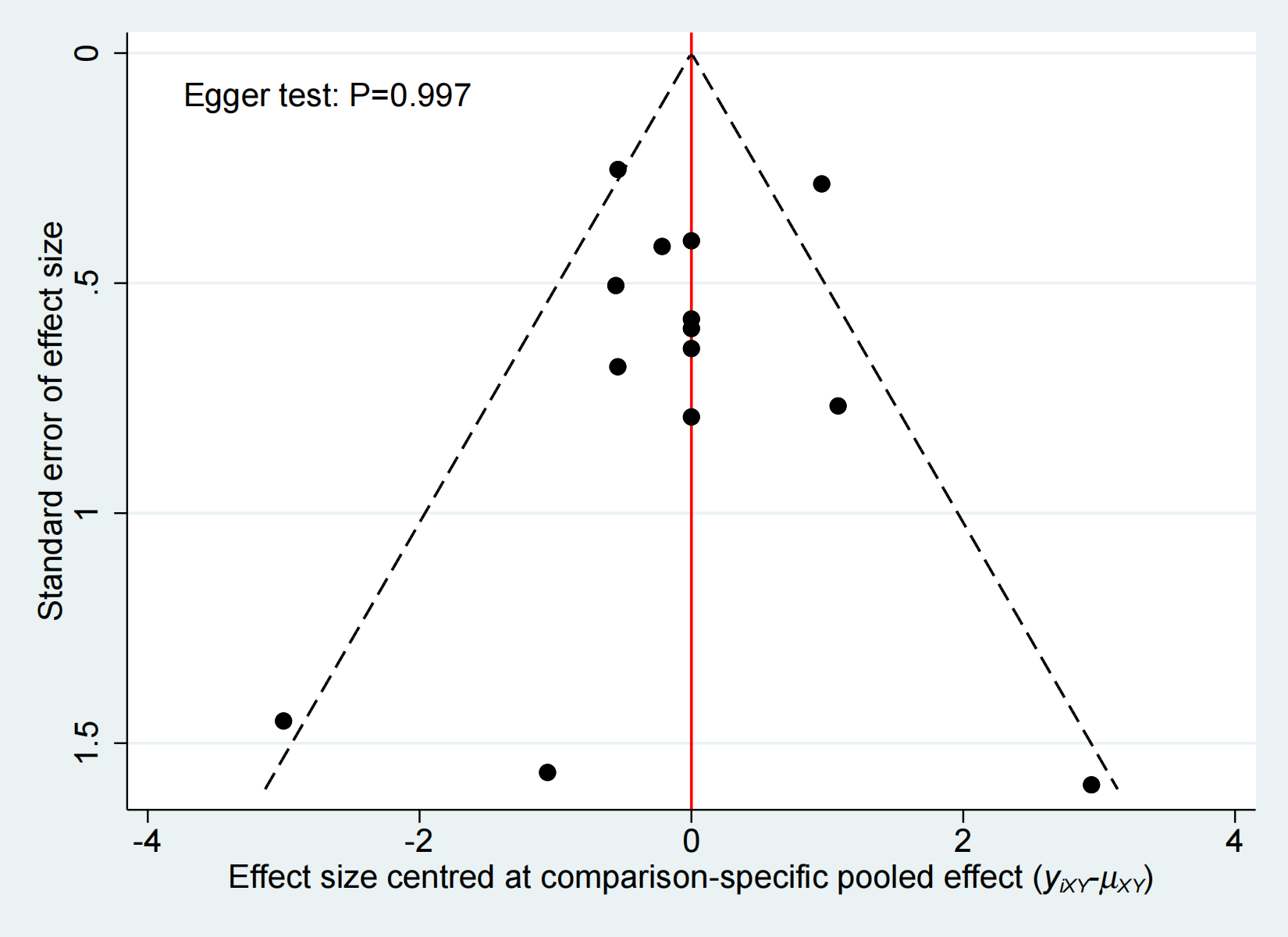


Figure 4.9 The funnel plot of Pre-eclampsia. The result of Egger test showed the p=0.997.


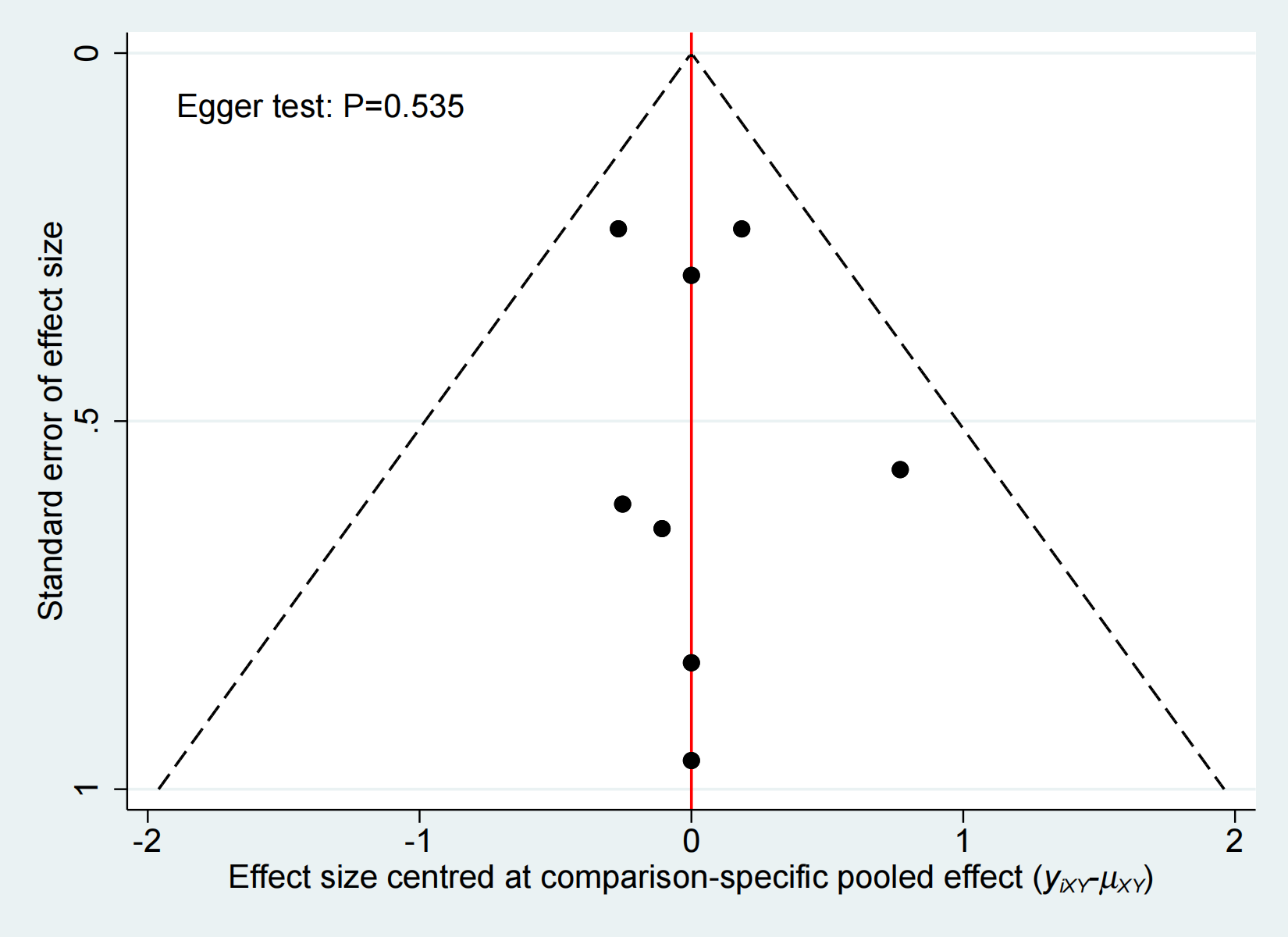


Figure 4.10 The funnel plot of Cesarean. The result of Egger test showed the p=0.535.
